# Recent advances in characterizing the immune microenvironment and biomarkers of endometrial carcinoma

**DOI:** 10.3389/fimmu.2026.1779211

**Published:** 2026-05-29

**Authors:** Jianhong Ma, Guang Yao, Wei Wang

**Affiliations:** 1 The Department of Obstetrics and Gynecology at the Affiliated Hospital of Gansu University of Chinese Medicine, Lanzhou, China; 2 The Department of Obstetrics and Gynecology at the First Hospital of Lanzhou University, Lanzhou, China

**Keywords:** biomarker, endometrial carcinoma, immune cell, immune microenvironment, prediction model, risk score

## Abstract

This study builds upon existing research achievements in bioinformatics to systematically screen a comprehensive set of biomarkers and prognostic models associated with immune infiltration regulation in the tumor microenvironment of endometrial carcinoma. It elucidates the predictive value of these markers and models as well as the oncogenic signaling pathways in which they are involved, thereby providing a theoretical foundation and molecular insights into the characteristics of tumor immune infiltration in endometrial carcinoma. Through a cross-study analysis, five immune-related markers and two gene families consistently reported across multiple investigations were identified. Their clinical prognostic significance was validated, and potential mechanisms underlying their roles in carcinogenesis were proposed. Furthermore, the functional implications of immune infiltrating cells on tumor behavior are summarized, offering valuable guidance for target identification and therapeutic strategy development in future clinical immunopharmacological research.

## Introduction

Uterine corpus endometrial carcinoma (UCEC), one of the most prevalent tumor diseases affecting women’s health and longevity worldwide, has consistently ranked as the leading gynecological malignancy in the United States in terms of incidence. Its mortality rate has shown a steady annual increase, emerging as a significant public health concern with implications for socioeconomic development. Since The Cancer Genome Atlas (TCGA) introduced the molecular classification of UCEC in 2013, researchers have employed high-throughput sequencing technologies over the past decade to investigate the oncogenic mechanisms and identify potential molecular targets across tumor subtypes, yielding substantial advancements. These developments are reflected in the updated FIGO staging criteria for UCEC released in 2023, which acknowledge that tumors with identical stages and histological types may exhibit distinct molecular and genomic profiles. The newly established risk stratification model integrates TCGA molecular subgroups with established clinical and pathological factors—such as myometrial invasion, histological subtype, and lymphovascular space invasion—signaling a transition into the era of precision medicine based on molecular profiling. In addition to conventional therapeutic approaches, immunotherapy has emerged as a pivotal component of systemic treatment. Individualized treatment strategies are now developed through a comprehensive evaluation of patients’ genetic and molecular data, aiming to achieve precise, long-term, and chronic management of the disease.

The integration of bioinformatics with high-throughput sequencing technology enables the effective identification and validation of key pathogenic genes as well as the prediction of disease risk. These insights can support personalized diagnostic and therapeutic strategies for patients with UCEC and facilitate the establishment of optimized follow-up schedules to minimize recurrence risk. Consequently, the discovery of novel prognostic biomarkers and therapeutic targets holds substantial scientific and clinical significance in improving the survival outcomes for UCEC patients. This study employed two innovative approaches to conduct an in-depth analysis of recent tumor sequencing data, reviewing both micro-level pathogenic mechanisms and macro-level genetic landscapes associated with UCEC and delineating immunological pathways critically involved in disease initiation and progression. Finally, the study established a mechanistic framework to support the rational development of future combination therapies incorporating both targeted agents and immunotherapeutics.

## Methods

A literature search was performed in the PubMed database to identify publications indexed between January 1, 2015 and December 31, 2025. The search strategy is detailed below: (“endometrial neoplasms”[MeSH Terms] OR “endometrial neoplasms”[Title/Abstract] OR “endometrial carcinoma”[Title/Abstract]) AND (“computational biology”[MeSH Terms] OR “computational biology”[Title/Abstract] OR “bioinformatic”[Title/Abstract] OR “bioinformatics”[Title/Abstract]) AND (“immunization”[MeSH Terms] OR “immunisations”[Title/Abstract] OR “immunizations”[Title/Abstract] OR “immunise”[Title/Abstract] OR “immunised”[Title/Abstract] OR “immuniser”[Title/Abstract] OR “immunisers”[Title/Abstract] OR “immunising”[Title/Abstract] OR “immunities”[Title/Abstract]). Duplicate records were removed, and titles/abstracts were screened for relevance. Following the full-text review, 241 articles satisfying all eligibility criteria were included. The inclusion criteria were as follows: (1) original research focused on endometrial cancer, (2) application of bioinformatics methodologies (e.g., differential expression analysis, pathway enrichment, machine learning, or multi-omics integration), and (3) explicit investigation of immune cell infiltration patterns within the endometrial tumor microenvironment.

## Identification and validation of immune-related genes

### Metabolic reprogramming

Metabolic reprogramming represents a fundamental hallmark of cancer, primarily manifested through enhanced glycolysis, increased glutamine utilization, and upregulated fatty acid synthesis. The capacity of cancer cells to survive, proliferate, and adapt within the nutrient-deprived tumor microenvironment (TME) relies heavily on metabolic reprogramming, which is intricately linked to immune regulation of tumor cells (1).

#### Glucose metabolism

Glycolysis represents the primary pathway of glucose metabolism in the human body and constitutes a key metabolic adaptation in tumor cells to meet the energetic and biosynthetic demands associated with rapid proliferation and differentiation. It supplies abundant ATP for tumor growth and fosters the TME that supports cell survival, invasion, and metastasis. Tumor cells exhibit markedly increased glucose uptake and rapidly generate ATP through the conversion of glucose into lactate even under aerobic conditions—a phenomenon known as the Warburg effect (2). Consequently, the heightened glycolytic activity observed in malignant tumor cells induces the formation of an acidic pericellular microenvironment, which promotes the demise of surrounding normal cells while accelerating tumor angiogenesis and invasive potential. A comprehensive investigation into the mechanisms and prognostic implications of glucose metabolic reprogramming in immune microenvironments is therefore of significant scientific and clinical importance in UCEC.

Lactate metabolism-related genes (LMRGs) were retrieved from a molecular feature database, and 18 LMRGs were selected to construct a prognostic risk model (**Supplementary Table 1**). The UCEC patients were stratified into high- and low-risk groups based on the median risk score, with the high-risk group significantly associated with poorer clinical outcomes. Functional enrichment analyses using GO and KEGG pathways revealed that LMRGs were prominently enriched in energy metabolism pathways and showed significant associations with tumor mutation burden (TMB), immune checkpoint expression, and immune cell infiltration. In the high-risk group, elevated infiltration levels of immune types C1, C2, and C4 were observed, along with increased activation of dendritic cells (DCs). In contrast, the low-risk group exhibited enhanced activation of plasma cells and FOXP3+ Tregs. These findings highlight distinct immunological characteristics within the UCEC microenvironment and provide valuable insights for advancing fundamental research (1). Hypoxia, as a key initiating factor in tumor metabolic reprogramming, disrupts glucose metabolism and reshapes the tumor immune microenvironment, promoting immune cell infiltration and extracellular acidosis. This adaptive response enables tumor cells to survive under hypoxic and nutrient-deprived conditions and contributes to resistance against radiotherapy and chemotherapy, ultimately leading to unfavorable clinical outcomes. Jiao developed a hypoxia-driven gene signature to investigate the relationship between the hypoxic TME and tumor recurrence, selecting four hypoxia-related differentially expressed gene (DEGs) as prognostic predictors and identifying two distinct molecular subtypes (3). Cluster 1 was characterized by lower tumor purity, suggesting a less immunosuppressive and more responsive TME, which correlated with improved prognosis. Based on these subtypes, it is recommended that patients in cluster 1 receive surgical intervention combined with targeted chemotherapy, whereas those in cluster 2 may benefit more from personalized immunotherapeutic approaches (3). Lin identified 10 GLRGs from the GeneCards database to establish a prognostic model for UCEC, stratifying patients into high- and low-risk groups (4). Overall survival (OS) was significantly lower in the high-risk group, and multivariate analysis confirmed that GLRGs serve as an independent prognostic factor. Unsupervised clustering analysis revealed two immune subtypes—IH and IC. The IH subtype displayed a reactive immunogenic phenotype, marked by substantial infiltration of anti-tumor immune cells, enrichment of pro-inflammatory cytokines, and coordinated expression of chemokines and related signaling molecules (4). Despite the presence of some pro-tumor elements, this robust immune activation likely contributes to the better prognosis observed in IH compared to IC (4). This study elucidates the unique biological features of the IH and IC subtypes, underscoring the potential of integrative classification strategies that combine molecular profiling with immunological characterization. These findings offer critical implications for future immunotherapy development in UCEC. Additionally, GZMM expression in tumor-infiltrating lymphocytes was identified as a potential biomarker of favorable prognosis (4).

In addition to aerobic glycolysis, mitochondrial oxidative phosphorylation (OXPHOS) is also closely associated with tumor progression. Liu developed an OXPHOS-related immune microenvironment prognostic model, in which patients with lower risk scores exhibited higher levels of immune cell infiltration, elevated ESTIMATE scores, increased immune phenotype score (IPS), microsatellite instability (MSI), and TMB. Conversely, these patients demonstrated lower tumor purity, reduced expression of mismatch repair (MMR) proteins, and decreased levels of m6A mRNA methylation regulatory factors. They also showed greater sensitivity to immune checkpoint blockade (ICB) and conventional chemotherapy agents, contributing to a more favorable prognosis (5). Nicotinamide adenine dinucleotide (NAD+) serves as a critical coenzyme in OXPHOS and is essential for various biological processes, including immune responses (6). During glycolysis, cytoplasmic NAD+ is regenerated via the lactate dehydrogenase (LDH) reaction, thereby supporting tumor growth. This metabolic adaptation results in an elevated NADP+/NADPH ratio and a higher NAD+/NADH ratio in cancer cells, underscoring the pivotal role of NAD+ in metabolic reprogramming. Hu constructed six NAD+-related gene signatures as predictive variables for prognostic stratification, wherein the high-risk group was significantly associated with poorer clinical outcomes (7). According to TME analyses, high-risk patients exhibited significantly reduced ESTIMATE scores, diminished enrichment of immune infiltrating cells, and downregulated expression of most immune checkpoint genes, indicating a higher likelihood of immune escape. These findings collectively reflect a profoundly immunosuppressive TME in high-risk individuals (7). Overall, these results suggest that NAD+ metabolism is intricately linked to the immune-genomic landscape of UCEC. Reduced immune cell infiltration in high-risk patients may contribute to adverse prognoses, implying that low-risk patients are more likely to benefit from immunotherapeutic interventions.

In summary, the glucose-metabolism-based risk model identified in the aforementioned research defines the novel molecular subtypes of UCEC and integrates them with clinical parameters, demonstrating significant potential for prognostic prediction and clinical management guidance—particularly in therapeutic decision-making regarding immunotherapy and chemotherapy.

#### Lipid metabolism

**Figure 1:**
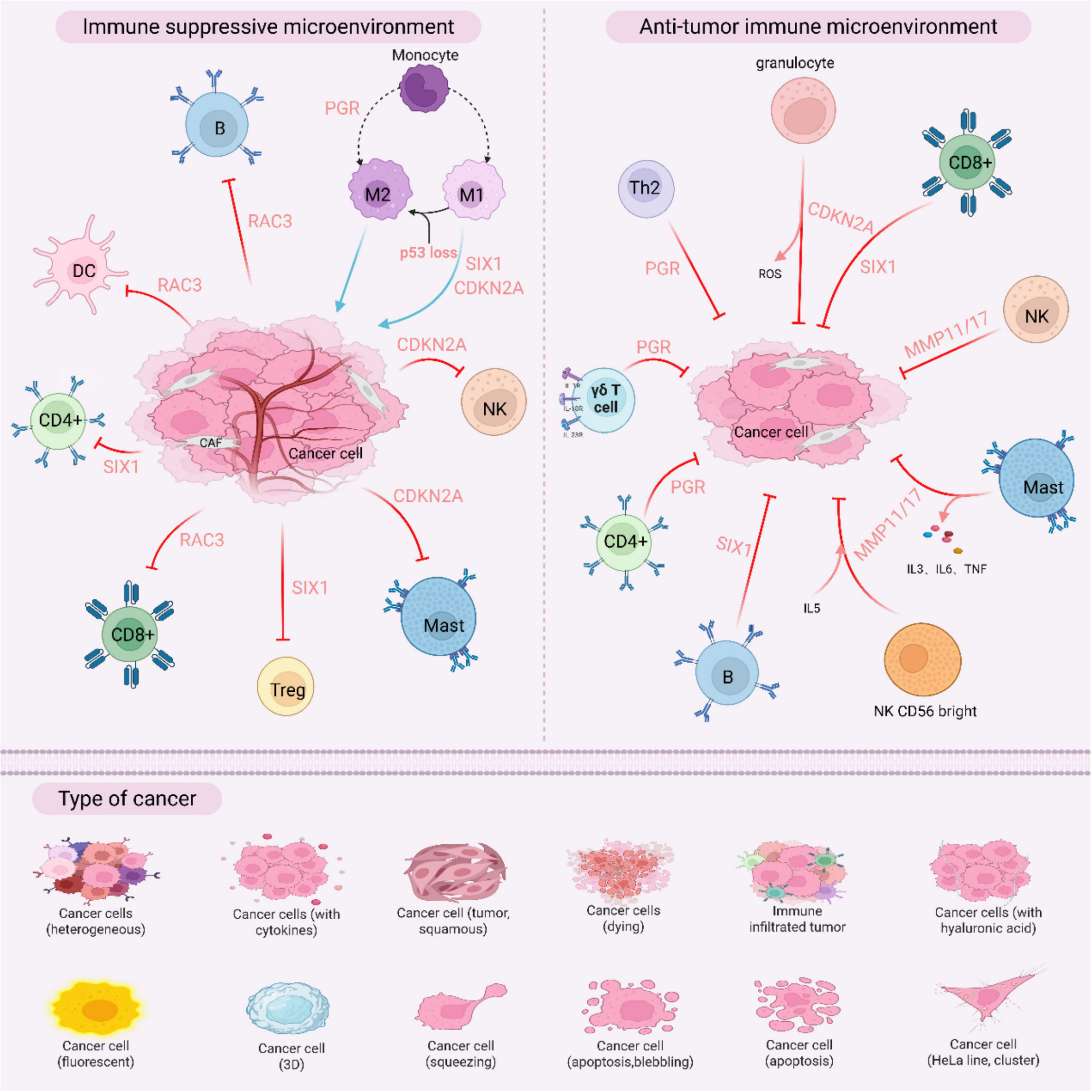
High-frequency overlapping genes and their association with the tumor microenvironment, including immunosuppressive and anti-tumor immune microenvironments.

Fatty acids—defined as molecules consisting of hydrocarbon chains terminated by a carboxyl group—are key constituents of lipids and participate in numerous biological processes. They promote tumor cell proliferation and migration by modulating membrane fluidity, inducing remodeling of the tumor microenvironment, and generating oncogenic signaling molecules. Reprogramming of fatty acid metabolism encompasses alterations in fatty acid uptake, *de novo* synthesis, post-synthetic modification, and β-oxidation and contributes to various metabolic adaptations across different stages of tumorigenesis (8) —for example, Cai demonstrated that RAC3 is upregulated in UCEC tissues and cell lines and promotes tumor cell proliferation and invasion through enhanced expression of fatty acid synthase (FASN). High RAC3 expression is significantly associated with poor prognosis and reduced immune cell infiltration in UCEC patients (9). During tumor progression, fatty acids not only facilitate cell migration and invasion but also stimulate angiogenesis through signal-mediated mechanisms, thereby contributing to immune evasion by tumor cells (**Figure 1**) (10–12). Moreover, fatty acid metabolic reprogramming influences the tumor immune microenvironment by modulating the abundance and functional activity of infiltrating immune cells, ultimately promoting immune escape (12) (**Figure 1**).

Li obtained transcriptome sequencing data from 552 UCEC patients in the TCGA database, extracted fatty acid metabolism-related genes from the KEGG database, and constructed a prognostic risk model. The results revealed significant differences in immune cell infiltration between high- and low-risk groups, with patients in the high-risk group exhibiting a more favorable response to immunotherapy (8). Yang integrated lipid metabolism and ferroptosis-associated gene (LMRGs-FARs) from the MSigDB and FerrDb databases, respectively, and developed a six-gene risk signature for UCEC. The low-risk group was positively associated with improved prognosis, high TMB, increased immune cell infiltration, higher expression levels of immune checkpoint molecules including CTLA4, GZMA, and PDCD1, and greater sensitivity to anti-PD-1 therapy (13). Additionally, Lin identified 10 GLRGs to construct a prognostic model for UCEC, which showed significant associations with immune cell infiltration, TMB, and chemotherapeutic sensitivity. This study provided valuable insights for future immunotherapeutic strategies in UCEC. Notably, GZMM expression in tumor-infiltrating lymphocytes was identified as a potential biomarker of favorable prognosis, a finding validated in independent clinical cohorts (4). In summary, these studies have established prognostic models linked to survival risk in UCEC patients, enabling the assessment of individual immune status and responsiveness to chemotherapy and targeted small-molecule therapies. Such models, along with associated molecular and phenotypic profiles, can assist clinicians in evaluating patient prognosis and tailoring optimal treatment strategies, thereby offering novel perspectives and potential therapeutic targets for personalized diagnosis and immunotherapy in UCEC.

#### Glutamine metabolism

γ-Glutamyl hydrolase (GGH) is a lysosomal enzyme that maintains intracellular folate homeostasis by cleaving glutamate residues and shortening the polyglutamate chains of MTXPGs, thereby facilitating the efflux of MTX from target cells and contributing to the development of treatment resistance (14). Zhu demonstrated that GGH enrichment is significantly associated with poorer OS, progression-free survival (PFS), and disease-specific survival (DSS) in UCEC (14). Furthermore, GGH expression is involved in regulating cancer cell proliferation and immune responses, showing a negative correlation with T cells, NK cells, and B cells. Overexpression of GGH correlates with significantly reduced TMB and lower TIDE scores, suggesting that patients in the low-GGH subgroup may derive greater benefit from ICI therapy for UCEC (14). Yu also investigated the expression level and prognostic significance of GGH in UCEC tissues, establishing associations with clinical characteristics and patient outcomes (15). Both mRNA and the protein expression levels of GGH were elevated in tumor tissues, and high GGH expression was significantly linked to advanced clinicopathological features and adverse prognosis (15). Functional analyses using GO, KEGG, and PPI networks revealed that GGH expression alterations are significantly enriched in pathways related to cell proliferation, immune regulation, and tumorigenesis in UCEC (15). Moreover, high GGH expression was associated with increased infiltration of Th2 cells and reduced levels of NK CD56bright cells (15). In summary, these findings indicate that GGH drives UCEC progression, serves as a promising biomarker and potential therapeutic target for survival prediction, and may impair immune cell infiltration and responsiveness to immunotherapy. Future studies should focus on elucidating the roles of associated signaling pathways in cancer cell proliferation and apoptosis.

### Programmed cell death patterns

#### Ferroptosis

Ferroptosis, a form of regulated cell death driven by iron-dependent lipid peroxidation, is closely associated with various endometrial disorders, including endometriosis (16), recurrent implantation failure (17), and endometrial hyperplasia (18). Accumulating studies suggest that tumor-associated inflammation often promotes immunosuppression in myeloid cells, redirecting hematopoiesis from steady-state conditions to emergency granulopoiesis, thereby impairing the generation of granulocytes, NK cells, and monocytes, ultimately facilitating tumor progression (19). The targeted induction of ferroptosis is increasingly recognized as a viable therapeutic strategy for overcoming resistance to conventional cancer treatments.

MGST1 is a key mediator of inflammatory processes and exhibits glutathione S-transferase and peroxidase activities localized to the endoplasmic reticulum and outer mitochondrial membrane, where it protects cellular membranes from oxidative-stress-induced damage and plays a critical role in suppressing iron-dependent cancer cell death, particularly ferroptosis (20). Yan aimed to elucidate the expression profile and prognostic significance of MGST1 in UCEC, thereby offering novel insights for theoretical understanding and ferroptosis-targeted immunotherapy. These findings suggest that MGST1 overexpression enables UCEC cells to effectively evade ferroptosis, leading to decreased infiltration of NK cells and CD8^+^ T cells while promoting increased accumulation of myeloid-derived suppressor cells (MDSCs) (21). NK cells and CD8^+^ T cells are key effector lymphocytes responsible for mediating anti-tumor immune responses and are highly responsive to alterations in the tumor microenvironment during cancer progression (21). This study indicates that elevated MGST1 expression is closely linked to tumor development, adverse prognosis, and dysregulated immune cell infiltration in UCEC. These insights could inform the future design of MGST1-targeted ferroptosis inducers and support the development of promising combination therapies for UCEC. Based on data from the TCGA database, Liu identified ferroptosis-related genes (FRGs), selected six prognosis-associated genes, and constructed a comprehensive overall survival prediction model for patients with UCEC. The results demonstrated that these FRGs were significantly associated with the tumor immune microenvironment, showing positive correlations with M1 macrophages, M2 macrophages, follicular helper T cells, and naïve B cells while exhibiting negative correlations with NK cell activation, Tregs, neutrophils, and resting DCs. This indicates that patients in the low-risk group exhibit enhanced immune activation and may be more responsive to immunotherapy, potentially deriving clinical benefit from such treatment (22). Additionally, Liu further analyzed 150 iron-related genes (IRGs) and selected six DEGs with prognostic significance to construct an independent risk model. The ssGSEA results showed significant differences in the infiltration levels of 16 immune cell types and the activity of 13 immune-related functions across risk groups (23).

By integrating FRGs with clinical factors, it is possible to predict patient responses to immunotherapy and chemotherapy, thereby providing valuable guidance for research on ferroptosis in UCEC and offering critical insights for the development of personalized treatment strategies.

#### Cuproptosis

**Figure 2:**
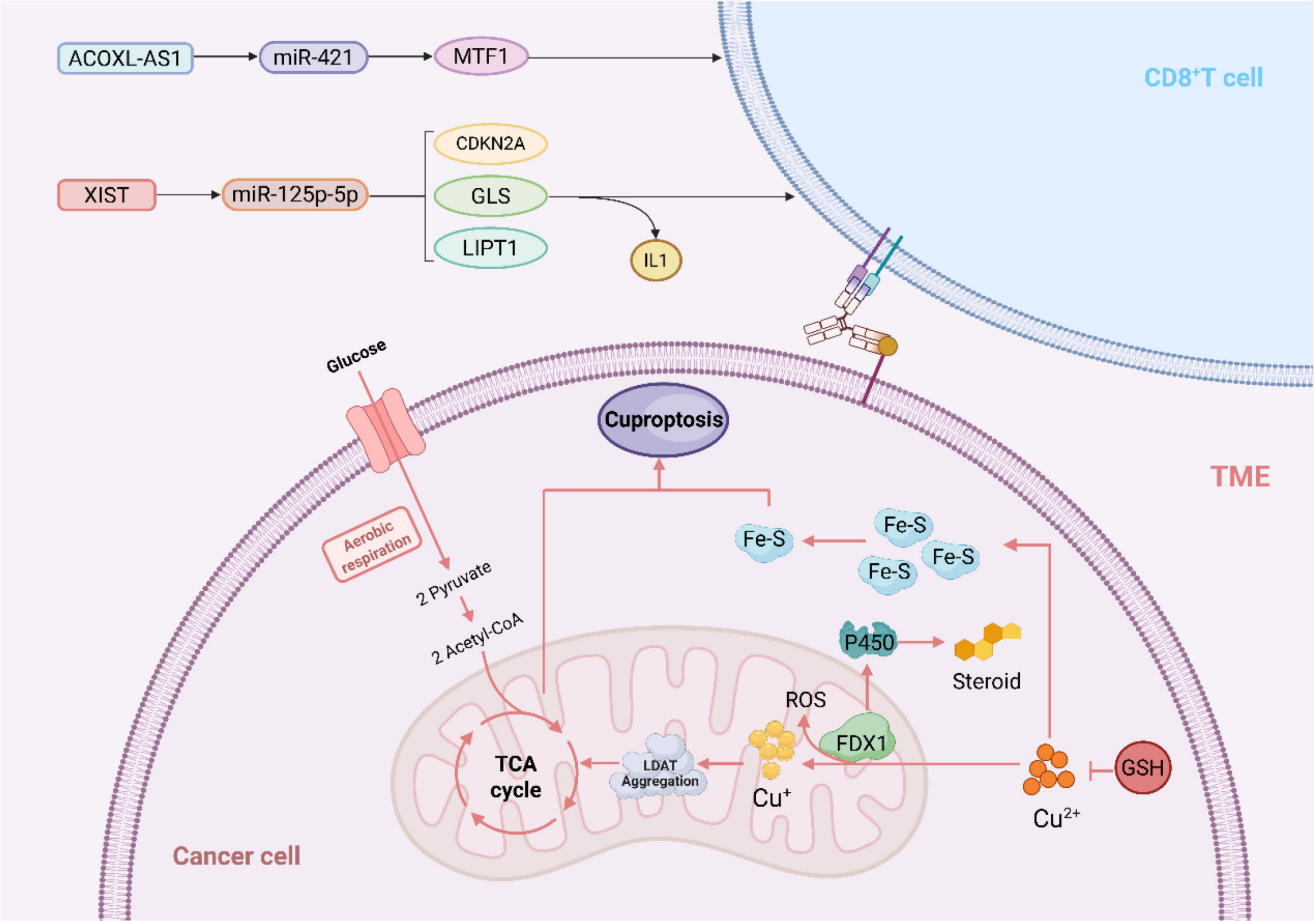
Ferroptosis and the TCA cycle.

It is noteworthy that cuproptosis, a form of regulated cell death mediated by copper ions, was first described by Tsvetkov in 2022 as a distinct mechanism of metal-ion-dependent mitochondrial cell death following the discovery of ferroptosis in 2012. Unlike ferroptosis, which primarily involves iron- or lipoxygenase-catalyzed lipid peroxidation of polyunsaturated fatty acids in cellular membranes, cuproptosis is closely linked to mitochondrial respiration, particularly through disruption of the mitochondrial TCA cycle (24). During cuproptosis, two major intracellular events occur: first, the accumulation of copper ions induces aberrant oligomerization of lipoylated proteins within the tricarboxylic acid cycle (TCA cycle) by direct binding; second, excessive copper leads to the depletion of Fe–S cluster proteins. These dual perturbations collectively trigger proteotoxic stress, ultimately resulting in cell death (24–26). Accumulating evidence indicates that, compared to healthy individuals, cancer patients exhibit elevated copper levels in both serum and tumor tissues. Excess copper can influence the metabolic activity of cancer cells as well as that of tumor-infiltrating immune cells (**Figure 2**).

FDX1 is a ferredoxin that facilitates the reduction of cytochrome P450 enzymes, thereby driving steroid biosynthesis and drug metabolism. As a direct molecular target of elesclomol, FDX1 also catalyzes the conversion of Cu²^+^ to Cu^+^—a more reactive and cytotoxic form—highlighting the therapeutic potential of targeting cuproptosis in oncology (**Figure 2**) (27). Elevated FDX1 expression has been shown to suppress ATP and lactate production while increasing ROS and glucose levels. FDX1 overexpression significantly inhibits UCEC cell proliferation and tumor growth, accompanied by downregulation of key proliferative markers including PCNA, HK2, PKM2, and Ki-67. These copper-related genes (CRGs) may serve as potential biomarkers and therapeutic targets for modulating immune cell infiltration within the TME of gynecological cancers (28). Another study confirmed a cuproptosis-related prognostic model comprising three potential biomarkers. Patients with a high expression of CDKN2A and GLS exhibited lower OS rates, while those with an elevated expression of CDKN2A, GLS, and LIPT1 showed poorer PFS (29). In immune infiltration analyses, the expression levels of these three prognostic genes were positively correlated with the abundance of immune cells such as CD8^+^ T cells and neutrophils. The ceRNA network further revealed the lncRNA XIST/miR-125a-5p/CDKN2A regulatory axis in UCEC, which may contribute to tumor progression, providing additional theoretical support for investigating the mechanisms of carcinogenesis and disease progression in UCEC (**Figure 2**) (29). Lin conducted a cuproptosis-related study identifying four differentially expressed CRGs to construct a prognostic prediction model in UCEC. Functional enrichment analysis indicated that this CRG-based model is involved in cell–cell adhesion processes and immune-related activities, including IL-1 signaling and cellular responses to cytokine stimulation. Further analysis demonstrated that the incidence of MSI-H was lower in the high-risk group compared to the low-risk group, and patients in the low-risk group displayed greater immune cell infiltration, suggesting enhanced responsiveness to immunotherapy (30). Collectively, these findings indicate that the CRG signature combined with dMMR status serves as a robust prognostic predictor in UCEC, offering new insights into immune microenvironment regulation and immunotherapeutic strategies. This evidence underscores the potential of cuproptosis modulation as a therapeutic approach for malignant tumors reliant on mitochondrial respiration or characterized by elevated copper levels.

lncRNAs are functional RNA molecules exceeding 200 nucleotides in transcript length that lack protein-coding capacity. Due to their cell- and tissue-specific expression patterns, lncRNAs play critical roles in regulating tumorigenesis, cancer progression, metastasis, drug resistance, tumor immune microenvironment, energy metabolism, histone modifications, and post-transcriptional regulatory processes. They have been increasingly recognized as potential diagnostic biomarkers and therapeutic targets in oncology. Hu identified two cuproptosis-related lncRNAs (CRLs) to construct and validate a novel prognostic model for UCEC (31). The risk score derived from this cuproptosis-associated model was significantly correlated with clinical and pathological molecular subtypes (31). Notably, substantial differences were observed between risk groups in immune function, immune checkpoint expression, and CD8^+^ T lymphocyte infiltration, indicating that the model may serve as an independent prognostic factor (31). Additionally, chemotherapy agents such as Akt inhibitors exhibited lower IC50 values in high-risk patients, suggesting enhanced drug sensitivity (31). Mechanistically, ACOXL-AS1 was proposed to function as an endogenous “sponge” that regulates MTF1 expression by sequestering miR-421 (**Figure 2**) (31). Furthermore, Zhang selected 12 to develop a risk model with excellent prognostic performance (32). Functional enrichment analysis revealed that these CRLs were predominantly involved in tumorigenesis and immune response pathways (32). Patients in the low TMB group showed poorer OS, highlighting the predictive value of CRLs for immunotherapy response and further validating the robustness of the prognostic signature (32). Moreover, Cai screened seven prognosis-associated cuproptosis-related lncRNAs to construct a risk model (33). The OS of patients in the low-risk group was significantly longer than that of the high-risk group. Furthermore, the low-risk group exhibited greater CD8^+^ T cell infiltration, a stronger IFN-II response, and increased sensitivity to cisplatin (33).

Based on these findings, it can be inferred that the CRL prognostic model may play a significant role in the pathogenesis and progression of UCEC. Its high reliability and accuracy in predicting clinical outcomes and response to immunotherapy provide valuable insights for investigating tumor molecular mechanisms and improving patient prognosis. Furthermore, this model elucidates the impact of CRLs on immune-related functions and immunotherapeutic responses in UCEC, offering a novel direction for guiding clinical risk stratification and optimizing individualized treatment strategies.

#### Oxidative-stress-induced apoptosis

Oxidative stress arises from an imbalance between the production of ROS and the cellular antioxidant defense capacity. Various cell types, including endometrial epithelial cells, oocytes, and vascular endothelial cells, are susceptible to the accumulation of persistent ROS. This redox imbalance can directly or indirectly disrupt intracellular signaling pathways, resulting in cellular and tissue damage that contributes to a range of disorders in the female reproductive system (34). In UCEC, a hallmark clinical symptom—irregular vaginal bleeding—leads to the release of large amounts of hemoglobin. The degradation products of hemoglobin, particularly free iron, promote oxidative stress through Fenton reactions, thus accelerating tumor initiation and progression (35).

SLC7A11 plays a critical role in maintaining intracellular glutathione levels and protecting cells against oxidative-stress-induced cell death. Research by Li demonstrates that SLC7A11 expression is significantly upregulated in patients with UCEC (36). Furthermore, SLC7A11 overexpression has been associated with tumor-infiltrating immune cells (TIICs), ICB, and response to immunotherapy, suggesting a favorable prognosis for UCEC patients with high SLC7A11 expression (36). In addition, Liu identified 136 oxidative-stress-related genes (OSRGs) from UCEC samples and constructed a risk score model based on seven prognostic genes selected through Lasso regression analysis, all of which showed a significant association with oxidative stress. This OSRG-based model was found to correlate with immune cell infiltration in UCEC, particularly CD8^+^ T cells, NK cells, and mast cells, with higher risk scores negatively correlated with immune activation (37). In the high-risk group, the expression of key immune checkpoint molecules—including CTLA4, TIM-3, TIGIT, and PD-1— was significantly downregulated, indicating that oxidative stress may influence UCEC progression through the modulation of immune cell activity (37). In summary, deeper insights into oxidative-stress-related biomarkers and the development of prognostic models highlight the potential of these genes and lncRNAs as novel biomarkers for predicting UCEC prognosis. These findings provide reliable biological indicators for clinical outcome assessment and offer new perspectives for immunotherapeutic strategies in UCEC.

#### Necroptosis

Necroptosis is a caspase-independent form of programmed cell death, the molecular core of which consists of effector proteins including RIPK1, RIPK3, and MLKL (38). This mode of cell death is mediated by a RIPK1/RIPK3-dependent phosphorylation signaling cascade and has been implicated in a wide range of human diseases, such as ischemic injury, neurodegenerative disorders, inflammatory conditions, and malignant tumors (39, 40). Notably, necroptotic cell death can be induced by specific features of the TME, and multiple miRNAs and lncRNAs serve as critical regulatory components by modulating necroptotic signaling and reshaping the TME. Therapeutic strategies targeting necroptosis and its regulatory networks in tumor cells may therefore represent a promising avenue for the treatment of UCEC.

It has been reported that He identified seven necroptosis-related lncRNAs (NRLs) to construct a prognostic risk model (41). ssGSEA revealed that, compared to the low-risk group, the high-risk group exhibited greater enrichment in immune- and tumor-related signaling pathways and biological processes (41). Notably, macrophage infiltration levels were higher in the high-risk group. Tumor-associated macrophages (TAMs) promote tumor cell invasion and extravasation while suppressing antigen presentation, thereby contributing to tumor progression and metastasis (41). Furthermore, the expression levels of immune checkpoint molecules were consistently lower in the high-risk group than in the low-risk group, suggesting that this subgroup may represent a non-responsive subtype to immune checkpoint inhibitor therapy. These findings provide mechanistic insights into the limited efficacy of immune checkpoint inhibitors in UCEC treatment (41). miRNAs are small non-coding RNA molecules that primarily regulate the expression of protein-coding genes by forming RNA-induced silencing complexes or through base-pairing with target mRNAs. Dysregulation of miRNAs has been implicated in the initiation and progression of malignant tumors. Zhang developed a novel necroptosis-related miRNA signature to predict UCEC prognosis, constructing a risk model based on two miRNAs-hsa-miR-425-5p and hsa-miR-7-5p—which were validated as independent prognostic factors for UCEC (42). Immune infiltration analysis demonstrated that the infiltration levels of CD8^+^ T cells, NK cells, and M1 macrophages were lower in tumor tissues compared to normal tissues (42). Furthermore, the necroptosis-related immune gene THRB was significantly associated with patient prognosis. Both WB and IHC analyses confirmed the differential expression of THRB between normal endometrial and tumor tissues (42). In conclusion, this study successfully identified and characterized novel necroptosis-related lncRNA and miRNA biomarkers, which may facilitate the application of precision medicine in clinical diagnosis and the development of immunotherapeutic strategies for UCEC.

### Endoplasmic reticulum stress

Within the TME, various adverse conditions—such as ischemia and hypoxia, nutrient deprivation, oxidative stress, intracellular calcium dysregulation, high metabolic demands, and DNA damage—can impair the ER’s protein processing capacity. This disruption leads to the excessive accumulation of misfolded or unfolded proteins, thereby inducing a cellular state known as “endoplasmic reticulum (ER) stress” (43). In response, the UPR is activated to restore ER homeostasis, which may result in cell reprogramming, adaptive survival, autophagy, or, under sustained stress, apoptotic cell death (44). Moreover, ER stress can reprogram immune cell functions, leading to the suppression of anti-tumor immunity (45). Specifically, ER-stressed tumor cells release soluble factors that alter the phenotype and function of surrounding leukocytes, promoting a pro-tumorigenic microenvironment (45). Furthermore, these stressed tumor cells upregulate PD-L1 expression and drive the M2 polarization of macrophages, facilitating immune evasion (45). Conversely, ER-stressed M1 macrophages secrete DAMPs, which can promote immunogenic tumor cell death (45).

Zhou extracted clinical and RNA sequencing data from 523 patients with UCEC in the TCGA database and identified four endoplasmic reticulum stress-related genes (ERGs) through LASSO Cox regression analysis to construct a prognostic risk model (45). The low-risk group exhibited higher infiltration levels of CD8^+^ T cells and Tregs, potentially contributing to better OS—indicating that greater TIIC abundance is associated with improved prognosis (45). Thus, this risk model may indirectly predict immunotherapeutic efficacy. Additionally, Zhang developed a four-ERG-based risk model, randomly assigning patients in a 1:1 ratio to training and validation cohorts and stratifying them into high- and low-risk groups based on risk scores, which effectively predicted immunoreactivity in UCEC patients (46). Survival analysis revealed that patients with higher risk scores had significantly worse outcomes, underscoring the clinical relevance of the model (46). Moreover, patients with higher risk scores demonstrated reduced immune cell infiltration, enabling immune evasion, along with lower expression levels of immune checkpoint molecules, further indicating the model’s utility in predicting immunotherapy response (46). In summary, these studies have established multiple ERG-related prognostic models capable of stratifying UCEC patients into distinct subgroups. These models facilitate risk stratification, enhance understanding of the interplay between ER stress-related genes and the TME, and reflect underlying immune activity. Future research will further validate their prognostic value and elucidate their molecular mechanisms using clinical specimens and *in vitro* experimental models.

#### Mitophagy

Mitophagy is a selective form of autophagy that functions as a key mitochondrial quality control mechanism by eliminating damaged or dysfunctional mitochondria under various cellular stress conditions, thereby reducing overall mitochondrial mass. It plays a critical role in the initiation, progression, and therapeutic response of malignant tumors, including UCEC. As mitophagy serves as an anti-apoptotic pathway in immune cells, it can modulate immune cell survival and function, thereby influencing tumor immune infiltration and patient prognosis.

TIMM8A is a protein-coding gene located on the X chromosome and a member of the mitochondrial intermembrane space chaperone network. It functions in a chaperone-like manner in complex with Tim13 to facilitate the import of nuclear-encoded precursor proteins into the inner mitochondrial membrane, playing a critical role in maintaining mitochondrial morphology and fission (47). Alterations in TIMM8A expression can lead to significant changes in mitochondrial structure and impair mitophagy. TIMM8A is upregulated in UCEC, where it inhibits cell proliferation, promotes immune cell apoptosis, and contributes to tumorigenesis—findings that may explain the strong negative correlation between TIMM8A expression and NK cell infiltration (47). Zhu reported that MDSCs and M2-polarized TAMs contribute to immune evasion by suppressing T cell activity (47). Aberrant TIMM8A expression may disrupt mitochondrial protein homeostasis in UCEC, thereby influencing immune cell infiltration and patient prognosis, particularly affecting CD8^+^ T cells, Th2 CD4^+^ T cells, and macrophage populations (47). Furthermore, TIMM8A shows promise as a predictive biomarker for anti-PD-L1 therapy efficacy, especially in strategies aimed at depleting MDSCs and M2 TAMs, thus offering valuable insights for future therapeutic development (47). In summary, these studies leverage mitophagy-related molecular signatures to predict UCEC prognosis, providing novel perspectives for survival prediction and personalized treatment selection and laying a foundation for optimizing anti-PD-L1 immunotherapy in clinical practice.

#### Oxidation–reduction process

Redox reactions play a central role in the initiation and progression of tumors and serve as effective modulators and therapeutic targets in anti-cancer treatment. The redox status of cancer cells represents a promising target for precision oncology, and prognostic models based on redox-related pathways may provide novel strategies for patient diagnosis and management. Redox-related genes (RRGs) were identified from public databases, and four RRGs were selected to construct molecular subclusters in UCEC (48). Significant differences in prognosis and immune activity were observed among these subclusters: patients in RRGcluster A exhibited milder clinical pathological features, higher levels of immune cell infiltration, elevated expression of immune checkpoint molecules, and improved survival rates; patients in RRGcluster B demonstrated the highest degree of immune infiltration, pronounced immune activation signatures, the highest TME score, and increased immune checkpoint expression, along with a greater likelihood of response to immunotherapy; patients in RRGcluster C had the shortest OS, the lowest TME score, and the highest tumor purity; and patients in RRGcluster D showed a distinct enrichment pattern compared to the other three clusters, with reduced activation in immune-related biological processes (48). Based on these findings, RRGcluster A and RRGcluster B are classified as “hot” tumors, characterized by robust anti-tumor immunity, while RRGcluster C and RRGcluster D resemble “cold” tumors, which are less likely to benefit from immunotherapy. A prognostic risk model based on RRGs was constructed using stepwise Cox regression analysis, identifying eight genes as potential biomarkers for predicting UCEC outcomes (48). The RRGs risk score was significantly associated with immune phenotype scores, MSI, TMB, tumor stemness index, CNV, and chemotherapy sensitivity (48). Compared to the high-risk group, patients in the low-risk group exhibited higher infiltration of activated CD8^+^ T cells, B cells, macrophages, monocytes, and Th17 helper cells, indicating enhanced anti-tumor immune capacity (48). Given that RRGcluster C and RRGcluster D—predominantly represented in the high-risk group—are classified as cold tumors, this suggests that individuals with high RRGs risk scores may exhibit impaired immune surveillance, facilitating tumor immune escape (48). Thus, the model demonstrates accurate assessment of clinical outcomes and possesses strong independent predictive power. Liu identified two key redox-related genes from the TCGA database to develop a prognostic model capable of predicting 3-, 5-, and 7-year survival probabilities (49). The results indicated that lower risk scores correlated with improved immunotherapeutic efficacy and better prediction of UCEC patients’ response to immunotherapy, thereby aiding in the identification of patient subgroups most likely to benefit from such treatments (49). Collectively, these studies have established RRG-based prognostic models that facilitate outcome evaluation and guide therapeutic decision-making. They comprehensively assess the impact of RRGs on the TME, clinical characteristics, and prognosis, highlight their value in targeted and immunotherapeutic strategies, and offer a new framework for advancing personalized treatment approaches in UCEC.

#### Bioactive molecule

##### Hormone

Although the proportion of estrogen/progesterone receptor-positive patients with UCEC is high, endocrine therapy remains effective in only a small subset of individuals. A deeper understanding of the molecular biology of estrogen/progesterone receptors may facilitate the identification of specific patient subgroups and support the development of novel therapeutic strategies. Elucidating the role of estrogen signaling in UCEC could provide critical insights for designing treatments that target or block the estrogen pathway. Li aimed to identify reliable prognostic markers among estrogen estrogen-response-related genes (DEERGs) and applied LASSO Cox regression analysis to screen 13 key candidate DEERGs, which were used to construct a prognostic model (50). This model demonstrated strong predictive performance and may serve as a potential independent indicator for assessing UCEC prognosis (50). The results indicated that patients in the low-risk group had significantly better clinical outcomes, increased immune cell infiltration, higher expression levels of HLA genes and immune checkpoint biomarkers, elevated TMB, and reduced copy number alterations—features collectively suggesting its potential value in predicting immunotherapy benefit (50). Low-risk patients were also more likely to respond favorably to anti-PD-1 immunotherapy. Wang’s comprehensive analysis revealed that both mRNA and protein expression levels of estrogen-related receptor α (ESRRA) are significantly upregulated in UCEC tissues (51). ESRRA contributes to tumorigenesis and disease progression by regulating cellular metabolic processes and is closely associated with poor prognosis (51). Immune infiltration analysis further indicated that ESRRA enhances neutrophil infiltration while reducing the abundance of CD4^+^ T cells, NK cells, and CAFs, suggesting a potential role in modulating the tumor immune microenvironment (51). Therefore, combining anti-estrogen agents with immunotherapy may represent a promising therapeutic strategy for UCEC patients (51). Collectively, these studies have systematically analyzed the expression patterns, clinical relevance, biological functions, immune landscape, and genomic alterations of estrogen-related markers in UCEC, providing a molecular foundation for future investigations into the functional mechanisms and therapeutic targeting of estrogen signaling pathways. As a potential systems-level approach to characterizing key determinants of UCEC pathogenesis and treatment response, improved understanding of ER and PR biology may help identify patient populations that are most likely to benefit from emerging therapeutic regimens.

Based on the shared SHMRGPI between breast cancer and UCEC, UCEC patients were stratified into high- and low-SHMRGPI groups (52). Patients in the low-SHMRGPI group exhibited longer OS and were characterized by higher infiltration levels of CD4^+^ T cells, γδ T cells, and Th2 cells (52). Given that CD4^+^ T cells and Th cells play essential supportive roles in anti-tumor immunity and that γδ T cells can eliminate tumor cells in a non-MHC-restricted manner, these findings reflect a robust anti-tumor immune response in the low-SHMRGPI group (52). Subsequent analyses revealed that multiple immune checkpoint genes, including PD-1, were more highly expressed in the low-SHMRGPI group, which also demonstrated a higher prevalence of MSI-H status and elevated TMB (52). Notably, the SHMRGPI values in MSI-H patients were significantly lower than those in MSS patients. The potential clinical efficacy of immunotherapy across SHMRGPI groups was assessed using the TIDE score, indicating that the high-SHMRGPI group was more susceptible to T cell dysfunction and immune evasion during immunotherapy (52). These results suggest that patients with low SHMRGPI may be more likely to benefit from both immunotherapy and targeted therapy (52). Therefore, this study systematically investigated the molecular characteristics of UCEC at genomic and transcriptomic levels, revealing that specific UCEC subgroups with distinct molecular profiles exhibit heightened sensitivity to targeted and immunotherapeutic interventions. These findings provide a theoretical foundation for guiding personalized treatment decisions and designing future precision medicine clinical trials.

##### Receptors and ligands

G protein-coupled receptors (GPCRs) represent one of the most extensively studied superfamilies of cell surface receptors, comprising over 800 members in the human genome. They play critical roles in diverse biological processes, including cell adhesion and motility, metabolic signal transduction, and immune responses. The aberrant expression of GPCRs has been frequently observed in various malignant tumors. Lei utilized multiple bioinformatics databases to investigate the expression patterns and potential functional implications of the adhesion GPCR family in UCEC. The results revealed that a significant association exists between adhesion GPCRs and tumor-infiltrating immune cells (53). ADGRA1, ADGRF1, and ADGRG3 are implicated in tumor-related inflammation and immune cell infiltration (53). Notably, ADGRC3 and ADGRF1 may serve as potential clinical biomarkers for UCEC, offering novel therapeutic targets for patient management (53). Chen’s study further demonstrated that integrating GPCR profiles with TME characteristics can inform clinical subclassification and optimize treatment strategies, particularly for combining GPCR-targeted approaches with immunotherapy in UCEC (54). Frequent TP53 mutations were identified in the GPCR-high/TME-low subgroup, which is enriched in CN–H molecular subtypes, suggesting enhanced proliferative capacity and poorer prognosis (54). In contrast, the GPCR-low/TME-high subgroup was more commonly associated with PTEN and ARID1A mutations, indicating potential links to distinct molecular subtypes (54). Collectively, these findings provide compelling evidence for exploring the expression landscape and functional relevance of GPCRs in UCEC. Characterizing the comprehensive interplay between GPCRs and immune cell populations within the TME may improve prognostic prediction and response assessment to immunotherapy, thereby enabling more precise clinical decision-making and advancing the role of GPCRs in cancer pharmacology.

##### Transcription factor

BHLHE22 is a member of the basic helix–loop–helix transcription factor family and plays a role in cellular differentiation (55). In UCEC, BHLHE22 protein expression is significantly downregulated (55). Darmawi reported a significant positive correlation between BHLHE22 expression and the infiltration of inflammatory TILs, including B cells, M1 macrophages, CD8^+^ T cells, CD4^+^ T cells, and myeloid dendritic cells, within the TME (55). Additionally, BHLHE22 expression shows a strong positive association with chemokines involved in recruiting macrophages and CD8^+^ T cells as well as with immune checkpoint molecules PD-1 and CTLA4 (55). These findings support the therapeutic potential of targeting the PD-1/PD-L1 and CTLA4 pathways, highlighting their promise as intervention targets in UCEC treatment (55). Furthermore, FOXM1, a member of the forkhead box superfamily, contains an evolutionarily conserved winged helix DNA-binding domain that underlies its function as a proliferation-associated transcription factor (56). The FOXM1 co-expression network indicates its involvement in immune cell infiltration, particularly through the induction of CD276 expression, which enhances neutrophil recruitment in UCEC (56). Moreover, FOXM1 knockout has been shown to inhibit UCEC cell proliferation, invasion, and migration (56). Lu’s systematic analysis revealed that IRF2, IRF3, IRF5, IRF6, IRF7, IRF8, and IRF9 are significantly overexpressed in UCEC, and alterations in their expression levels contribute critically to tumorigenesis and disease progression (57). The pathway analyses indicate that IRFs and their co-expressed genes are enriched in the PD-1/PD-L1 checkpoint pathway, further underscoring the potential utility of PD-1/PD-L1 inhibitors in UCEC therapy and providing deeper insights into the molecular heterogeneity and complexity of this malignancy (57). Collectively, this study confirms that multiple transcriptional regulatory factors are implicated in modulating immune-related signaling pathways and shaping the tumor immune microenvironment, suggesting their potential as predictive markers for ICI response, indicators of favorable prognosis, and therapeutic targets for immunotherapy in UCEC.

##### Biocatalytic enzymes

Biocatalytic enzymes, as key bioactive molecules essential for maintaining human physiological and metabolic processes, catalyze a wide range of enzymatic reactions in the body and play a critical role in modulating the TME in malignant cancers. Cai demonstrated that RAC3 expression is inversely correlated with the infiltration levels of B cells, CD8^+^ T cells, macrophages, and DCs, indicating its involvement in immune regulation within the UCEC tumor microenvironment (9). Furthermore, RAC3 expression was found to be negatively associated with monocyte markers (CD86, CD115), TAM markers (CCL2), and M1 macrophage markers (COX2), suggesting a pivotal role in TAM polarization (9). Additionally, RAC3 expression correlates with Th cell markers, including Th1-associated molecules (T-bet, STAT4), implicating it in T-cell-mediated immune modulation (9). Notably, the expression of key immune checkpoint molecules—PD-1, CTLA4, TIM-3, and GZMB—is negatively correlated with RAC3 levels (9). RAC3 expression is also significantly associated with molecular subtypes and the four consensus immune subtypes (C1–C4) (9). Mechanistically, RAC3 promotes tumor cell proliferation and invasion by upregulating FASN expression, highlighting its potential as a novel prognostic biomarker in UCEC (9). Another study further confirmed that RAC3 is specifically expressed in UCEC tumor cells, where elevated RAC3 levels are negatively correlated with CD8^+^ T cell infiltration and contribute to the establishment of an immunosuppressive microenvironment (58). Moreover, RAC3 accelerates tumor cell proliferation and suppresses apoptosis without altering cell cycle phase distribution. Silencing RAC3 enhances the sensitivity of UCEC cells to chemotherapeutic agents (58). Collectively, these findings indicate that RAC3 functions as an oncogene in UCEC, elucidating its potential mechanisms in tumor progression and immune evasion. These results suggest that RAC3 may serve as a promising biomarker for assessing the immune landscape of UCEC and could support the clinical development and application of immunotherapeutic strategies (**Figure 1**).

Enzymes involved in human metabolic processes also regulate the tumor immune microenvironment in UCEC, including MTHFD2 (59), PHGDH (60), and PSAT1 (61), which are upregulated in UCEC and strongly associated with poor prognosis. These enzymes may serve as independent prognostic biomarkers for patients with this disease. As a key enzyme in folate-mediated one-carbon metabolism, MTHFD2 catalyzes the production of S-adenosylmethionine, nucleotides, and amino acids such as serine and glycine—molecules essential for supporting malignant tumor growth (59). In UCEC, the SNHG3/hsa-miR-455-5p axis mediates the overexpression of MTHFD2, and MTHFD2 expression is closely linked to tumor immune infiltration and disease progression (59). It shows a significant positive correlation with increased immune cell infiltration, particularly with Th2 cells, Tcm, Th cells, Tgd, and macrophages, while exhibiting negative correlations with NK CD56bright cells, pDCs, NK cells, iDCs, and mast cells (59). Furthermore, MTHFD2 expression is significantly correlated with immune checkpoint genes such as CD274, HAVCR2, TIGIT, LAG3, and PDCD1LG2 in UCEC, contributing to an immunosuppressive microenvironment and worse clinical outcomes (59). PHGDH, the rate-limiting enzyme in the serine biosynthesis pathway, is highly expressed in UCEC and has been shown to reduce CD8^+^ T cell infiltration (60). PSAT1 plays a critical role in regulating UCEC cell proliferation, immune responses, and cell cycle progression (60). Its expression is positively correlated with Th2 cells but negatively associated with Th17 cells (60). Studies have demonstrated that miR-195-5p negatively regulates PSAT1 expression in UCEC, and the knockdown of PSAT1 suppresses cellular proliferation, migration, and invasion *in vitro* (61). Collectively, these findings indicate that metabolic enzymes influence UCEC prognosis, at least in part, through the modulation of immune infiltration. Targeting these catalytic enzymes may represent a promising strategy to counteract tumor immune evasion and disease progression, offering valuable predictive biomarkers and novel therapeutic opportunities for the diagnosis and treatment of UCEC.

Matrix metalloproteinases (MMPs) constitute a multigene family of zinc-dependent endopeptidases that regulate the biological activity of various cytokines and play pivotal roles in extracellular matrix remodeling. In UCEC, the elevated expression of MMP11 is associated with improved OS, whereas the reduced expression of MMP17 correlates with poorer OS outcomes (62). Furthermore, MMP11 and MMP17 expression levels exhibit significant correlations with immune cell infiltration. Tumors exhibiting a high expression of MMP11/17 demonstrate enhanced infiltration of NK cells, mast cells, and the NK CD56bright subset (62). NK cells predominantly infiltrate the tumor stroma and exert anti-tumor effects through direct interactions with tumor cells or modulation of other immune cells (62). Their presence within the TME has been linked to favorable clinical outcomes (63, 64). Mast cells secrete a range of cytokines, including IL-3, IL-6, and TNF. TNF inhibits endothelial cell proliferation, induces tumor tissue necrosis, and suppresses malignant metastasis by blocking angiogenesis (65). Consequently, mast cell infiltration in tumor tissues is associated with anti-tumor and host-protective functions (65). The NK CD56bright subset is typically characterized as immunoregulatory and can be activated by cytokines such as IL-5 (62). These cells possess cytotoxic capabilities and contribute to the enhancement of anti-tumor immunity (62). Therefore, a high expression of MMP11 and MMP17 may influence UCEC prognosis by modulating immune cell infiltration, and they could serve as effective prognostic biomarkers and potential therapeutic targets. Su developed an MMP score based on MMP-related DEGs. The expression pattern characterized by MMP cluster B is associated with a higher MMP score, greater abundance of immune cell infiltration in the TME, and stronger anti-tumor immune responses (66). This indicates that the MMP score serves as a reliable and robust tool for comprehensively assessing individual tumor-specific MMP expression profiles and can be used to determine the immune infiltration pattern, thereby defining the tumor immune phenotype (66). Given that the ECM can either remain confined to the tumor capsule or penetrate into the tumor parenchyma—facilitating intratumoral immune cell entry— MMP cluster A exhibits a pronounced state of matrix activation (66). Integrating the TME infiltration characteristics across clusters, it is proposed that matrix activation in MMP cluster A creates a physical or biochemical barrier that restricts immune cell function, thereby attenuating anti-tumor immunity and contributing to poor prognosis (66). This supports the validity of immune phenotyping based on distinct MMP expression patterns (66). Collectively, the MMP score holds promise as a multifaceted tool for evaluating immune infiltration status and survival outcomes in UCEC. It may provide more precise guidance for selecting immunotherapeutic strategies and open new avenues for epigenetic research in UCEC, ultimately facilitating the development of novel drug combinations or personalized immunotherapies.

#### Malignant biological properties

##### Proliferation- and metastasis-related

In recent years, bioinformatics technologies have identified multiple biomarkers associated with the malignant biological phenotypes of UCEC. Among these, proliferation- and metastasis-related molecular targets include MCM4 (67), MAL (68), SIX1 (69), CTHRC1 (70), CLCN4 (71), SPAG5 (72), ZDHHC1 (73), ZBTB7A (74), and DLC1 (75). The first six genes are overexpressed in UCEC, whereas the latter three exhibit downregulated expression. Alterations in the expression levels of these genes in tumor tissues have been consistently linked to poor patient prognosis. These molecules function through multiple oncogenic pathways and serve as independent prognostic predictors for UCEC. Immune infiltration analysis has demonstrated that high MCM4 expression is associated with reduced infiltration of T cells and B cells and positively correlates with PD-L1 expression. Elevated MCM4 levels are observed in the CN–H molecular subtype and the interferon-gamma-dominant (C2) immune subtype (67). Functional studies have confirmed that MCM4 overexpression promotes tumor cell growth and invasion while inhibiting apoptosis, thereby contributing to adverse clinical outcomes (67). Li has shown that MAL promotes UCEC cell proliferation by modulating cell cycle progression and suppressing differentiation and apoptosis (68). MAL expression is positively correlated with resting memory CD4^+^ T cells and immune checkpoint molecules such as CD274, LAG3, and PDCD1LG2 (68). Zhao’s immune infiltration analysis revealed that SIX1 expression is positively associated with tumor purity, B cells, and CD8^+^ T cells but negatively correlated with memory CD4^+^ T cells, Tregs, and neutrophil infiltration (69) (**Figure 1**). Research by Li demonstrated that CTHRC1 promotes the migration, invasion, and adhesion of UCEC cells via interaction with the integrin β3-Akt signaling pathway and upregulates the chemokine receptor CX3CR1, enhancing the recruitment of M2-polarized TAMs *in vitro*, thereby promoting tumor dissemination (70). Wang reported that CLCN4 expression is significantly correlated with CD4^+^ T cell infiltration, particularly Th1 subset infiltration. Further evidence indicates that CLCN4 downregulation exerts anti-tumor effects in UCEC (71). Mechanistically, CLCN4 functions as a Cl^-^/H^+^ antiporter involved in ion exchange. In tumor cells, where intracellular proton accumulation is elevated, efficient proton extrusion is critical for maintaining pH homeostasis, which may facilitate invasion by regulating endosomal pH (71). SPAG5 influences the infiltration levels of tumor-infiltrating immune cells in the TIME, as well as the expression of immune checkpoints such as LAG3 and TIGIT (T cell immunoreceptor with Ig and ITIM domains), potentially affecting immunotherapeutic response in UCEC patients and indicating its regulatory role in driving UCEC progression through the modulation of malignant cellular behaviors (72). Jiang demonstrated that high ZDHHC1 expression suppresses UCEC cell growth, proliferation, and cell cycle transition, likely through the inhibition of key oncogenic pathways including cell cycle regulation, DNA replication, and PI3K-Akt signaling (73). Mechanistic investigations reveal that ZDHHC1 expression is significantly correlated with RNA modification processes and alterations in tumor immune cell populations, including NK CD56bright cells, eosinophils, and Th2 cells, as well as various immune cell markers (73). Geng found that ZBTB7A exhibits strong positive correlations with multiple immune cell types, including neutrophils, DCs, T cells, Th1, Th2, and Tregs, indicating its role in modulating immune infiltration (74). *In vitro*, ZBTB7A was shown to inhibit the migration and invasion of Ishikawa cells by attenuating E2F4-mediated transcription (74). DLC1 functions as a GAP, promoting the conversion of active Rho-GTP to inactive Rho-GDP, thereby regulating cytoskeletal dynamics and tumor cell motility (75). CIBERSORT analysis indicates that resting memory CD4^+^ T cells and resting mast cells are positively correlated with DLC1 expression, whereas M2 macrophages show a negative correlation, highlighting DLC1’s pivotal role in shaping the immune landscape in UCEC (75).

In summary, these biomarkers may promote the proliferation and metastasis of UCEC cells by suppressing the activity of anti-tumor immune cells and reshaping the TME to facilitate immune tolerance or enable cancer cell immune escape. A comprehensive understanding of the biological functions of key genes involved in tumor progression and clinical outcomes is essential for elucidating their underlying molecular mechanisms. Further investigation and functional characterization of these molecules will not only enhance mechanistic insights but also offer a solid theoretical basis and identify promising therapeutic targets for the development of novel treatment strategies in UCEC.

##### Epithelial–mesenchymal transition

Epithelial–mesenchymal transition (EMT) is a dynamic and reversible biological process in which tumor cells transition from an epithelial to a mesenchymal phenotype. This transformation is characterized by the loss of cellular polarity and disruption of epithelial barrier integrity, enabling cells to acquire enhanced motility and invasive capabilities. Through the activation of EMT-related signaling pathways, these cells gain stem-cell-like properties, exhibit reduced cell polarity, and display weakened cell–cell adhesion, collectively promoting migratory and invasive behavior. Tumor cells exhibiting these phenotypic changes acquire heightened metastatic potential and are considered to undergo one of the key mechanisms underlying cancer invasion and dissemination.

A total of 10 EMT-related genes (ERGs) associated with the prognosis of UCEC were identified from 1,316 ERGs, and a predictive prognostic model was constructed and validated based on gene expression. GSEA was conducted to investigate the biological functions of this 10-ERG signature, revealing its significant association with the tumor immune microenvironment in UCEC (76). Specifically, the low-risk group exhibited enrichment in immune-related pathways, characterized by higher infiltration levels of CD8^+^ T cells, Tregs, and plasma cells, along with enhanced immune activity, elevated TMB, increased expression of CTLA-4 and PD-1, and a higher IPS (76). Furthermore, dMMR status was found to be associated with PD-L1 positivity and elevated CD8^+^ T cell infiltration, suggesting that this subgroup may benefit more from ICI therapy (76). In contrast, the high-risk group demonstrated activation of tumorigenesis-related pathways and greater infiltration of macrophages and follicular helper T cells, which correlated with poorer clinical outcomes (76). Ruan applied machine learning algorithms to identify nine ERGs as independent prognostic predictors (77). Immune infiltration analysis indicated that the high-risk group had lower M2 macrophage infiltration and relatively higher M1 macrophage presence, suggesting an improved cytotoxic T cell function and potential responsiveness to CTLA-4/PD-1 blockade therapy (77). The studies described above have established several EMT-related molecular signatures as novel classification tools for patients with UCEC. Compared to existing prognostic indicators, these newly developed models demonstrate greater potential for clinical application. Furthermore, they may serve as valuable references for predicting individualized responses to immune checkpoint inhibitors and chemotherapy agents.

##### Tumor angiogenesis

The ANGPT1 gene encodes a secreted glycoprotein belonging to the angiopoietin family, which binds to the endothelial cell membrane receptor tyrosine kinase TIE2 (also known as TEK), thereby promoting tumor angiogenesis. In UCEC, ANGPT1 expression is downregulated (78). Furthermore, ANGPT1 expression is negatively correlated with Tregs and activated NK cells while positively associated with resting memory CD4^+^ T cells, activated DCs, and activated memory CD4^+^ T cells (78). Higher ANGPT1 levels may enhance T cell infiltration in patients, contributing to improved prognosis and better OS (78). ATAD2 is an ATPase protein that is overexpressed in UCEC (79). Basic experimental evidence has demonstrated that ATAD2 upregulates VEGF expression, thereby promoting tumor growth and angiogenesis, and serves as an independent predictor of poor prognosis (79). The pathway enrichment analyses indicate that ATAD2 is involved in immune infiltration processes in UCEC, highlighting its potential as both a diagnostic biomarker and a therapeutic target (79). In summary, this study identifies ANGPT1 and ATAD2 as two tumor-angiogenesis-related molecules that function as diagnostic markers and potential therapeutic targets. These molecules may serve as early warning signals for malignant progression and could aid in the early screening of UCEC patients.

#### DNA mismatch repair system

Dysfunction of the DNA mismatch repair system is a primary driver of tumorigenesis. The MMR system comprises multiple genes responsible for recognizing and correcting DNA mismatches, a process essential for maintaining genomic stability. Impairment of MMR function compromises or abolishes DNA proofreading capacity, leading to the accumulation of replication errors and genomic instability, particularly MSI (80). MSH2 is a core component of the evolutionarily conserved MMR machinery, preserving genetic integrity by detecting DNA mismatches (80). When MSH2 undergoes mutation or deletion, its functional activity is disrupted, resulting in increased replication errors and ultimately contributing to phenotypic alterations (80). ssGSEA results demonstrate that MSH2 expression is positively correlated with the infiltration levels of Th2 cells, Tcm, and Tgd while negatively correlated with the infiltration of NK CD56 bright cells, plasmacytoid pDCs, immature iDCs, cytotoxic cells, and neutrophils (80). These associations suggest that MSH2 may facilitate immune evasion by promoting Th2 cell infiltration, highlighting its potential as both a diagnostic and prognostic biomarker and an immunotherapeutic target in UCEC (80). Additionally, gene enrichment analysis in the MSH6 low-expression group reveals predominant involvement in immune response regulation, which correlates with less aggressive disease features, higher immune cell infiltration—particularly CD8^+^ T cells—and increased expression of immune checkpoints, potentially conferring greater responsiveness to ICIs (81). Therefore, downregulation of MSH6 may serve as a promising predictive marker for favorable prognosis and enhanced therapeutic response to ICIs.

Uncontrolled cell proliferation is closely associated with the accumulation of extensive DNA damage and genomic instability. Efficient DDR mechanisms are essential for maintaining genomic integrity and preventing tumorigenesis. A novel risk model was developed based on nine differentially expressed DDR-related long non-coding RNA (DRLs) (82). In the high-risk subgroup characterized by dysregulated DDR activity, infiltration of most immune cell types was reduced, immune checkpoint molecules such as PD-1 and CTLA-4 were downregulated, and the majority of immune-related signaling pathways showed decreased activity, indicative of a “cold tumor” immune microenvironment (82). This suggests that patients in this group are less likely to benefit from ICB therapy and may exhibit resistance to radiotherapy and chemotherapy, along with reduced sensitivity to conventional chemotherapeutic agents and targeted inhibitors (82). In conclusion, targeting the DDR pathway to induce DNA damage-mediated immune responses represents a promising strategy for eliminating cancer cells. Given the potential resistance to radiotherapy, chemotherapy, and immunotherapy in high-risk patients, DDR inhibitors may serve as valuable therapeutic agents to improve clinical outcomes. Integrating DDR signaling with lncRNA regulatory networks may provide novel insights and innovative approaches for enhancing the clinical management of UCEC.

The efficacy of radiotherapy primarily depends on its capacity to induce DNA damage and trigger tumor cell death, which constitutes a critical determinant of radiotherapy response. Yang developed a risk model based on four DNA repair-related genes and classified patients into radiotherapy-sensitive (RS) and radiotherapy-resistant (RR) groups using a radiosensitivity index (83). The RS group showed significantly improved OS and DSS rates compared to the RR group (83). Notably, the RS group exhibited a higher proportion of naïve B cells and Tfh, while the RR group displayed elevated levels of Tregs and memory B cells (83). The OS rate in the RS- Tfh-high subgroup was markedly superior to that of other subgroups, suggesting that an increased presence of Tfh cells plays a pivotal role in promoting B cell activation and antibody production (83). In contrast, the RR-Tregs-low subgroup demonstrated significantly worse survival outcomes (83). An immunosuppressive TME characterized by Treg infiltration may contribute to radiotherapy resistance and poorer prognosis in UCEC patients (83). Furthermore, patients in the RS-PD- L1-high subgroup showed enrichment of immune-related pathways, enhanced immune cell infiltration, and greater responsiveness to immunotherapy, along with improved OS rates (83). This subgroup likely exhibits a more active and diverse anti-tumor immune response (83). Both intrinsic radiosensitivity features and PD-L1 expression status may serve as complementary predictive factors for radiotherapy response, potentially aiding in prognostic stratification and treatment optimization (83). These findings enhance our understanding of how to optimally integrate radiotherapy with other therapeutic modalities to improve clinical outcomes. Targeting Tregs or modulating the immunosuppressive TME may represent promising strategies to overcome treatment resistance and enhance the efficacy of immunotherapy, thereby enabling more precise and effective therapeutic interventions.

#### Epigenetic modification

Epigenetics refers to heritable changes in gene activity that occur without alterations to the underlying DNA sequence. Chromatin regulatory factors (CRs) are key upstream regulators in epigenetic processes, primarily mediating epigenetic modifications through three major mechanisms: DNA methylation, chromatin remodeling, and histone modifications—including phosphorylation, ubiquitination, SUMOylation, and acetylation. CRs function bidirectionally by both encoding and decoding chemical marks on cytosine residues and histone proteins, such as methylation and demethylation events. Additionally, chromatin remodelers can disrupt the interaction between nucleosomes and DNA, leading to nucleosome repositioning and, consequently, aberrant epigenetic states. Concurrently, epigenetic modifications significantly influence gene activity and cellular functions, thereby contributing to cancer progression —for instance, during epigenetic reprogramming, tumor cells may acquire immune tolerance, allowing them to evade immune surveillance and establish an immunosuppressive microenvironment.

##### DNA methylation

DNA methylation is a prevalent epigenetic modification that can activate or silence specific genes, thereby modulating downstream signaling pathways. Aberrant DNA methylation—encompassing both hypermethylation and hypomethylation—is closely associated with the development of various diseases, including UCEC —for example, Liu demonstrated that hypermethylation of the RILPL2 promoter contributes to its downregulation in UCEC (84). RILPL2 regulates the immune infiltration of various TIICs within the tumor immune microenvironment by influencing cell cycle progression, thereby directly or indirectly impairing immune surveillance and ultimately leading to poor clinical outcomes (84). This finding was further corroborated by studies on SYTL1 DNA methylation and its association with prognosis in UCEC patients (85). Additionally, Liao proposed that hypomethylation of the SPC25 promoter in high-grade UCEC results in the overexpression of SPC25 at both mRNA and protein levels (86). This upregulation suppresses the expression of chemokines and their receptors, thereby inhibiting the recruitment of key immune effector cells to the tumor site, attenuating anti-tumor immune responses, and contributing to disease progression (86). Collectively, these findings suggest that aberrant methylation patterns may serve as indicators of tumor immune infiltration and potential predictors of immunotherapy response in UCEC patients. Such epigenetic alterations may reshape the composition and functional state of the TME and disrupt immune processes, thereby altering anti-tumor immunity and influencing disease trajectory and outcomes.

DNA methylation is a distinct epigenetic modification that primarily involves the addition of a methyl group to cytosine residues in DNA. Other forms of nucleic acid modifications, such as RNA modifications including m6A, m1A, m7G, and m5C, represent separate regulatory mechanisms and will be discussed in detail in the following section.

##### N6-methyladenosine modification

Emerging evidence indicates that epigenetics plays a pivotal role in tumor initiation and progression by driving aberrant transcriptional programs. Tumor immunogenicity and the function of immune cells involved in anti-tumor immune responses can also be modulated by epigenomic alterations. Following RNA transcription, RNA molecules are subject to over 170 distinct post-transcriptional modifications; among these, m6A is the most prevalent, abundant, and evolutionarily conserved internal mRNA modification. The m6A modification governs nearly all aspects of RNA metabolism, including splicing, processing, nuclear export, translation, and degradation, thereby influencing gene expression and fundamental cellular processes. Moreover, m6A modifications have been shown to regulate immune cell activation and the infiltration of immune cells into the TME, thus impacting the efficacy of immunotherapy.

Pang developed a prognostic model based on the expression of two m6A regulators, IGF2BP1 and YTHDF3, which stratified UCEC patients into high- and low-risk groups (87). Compared to the low-risk group, the high-risk group exhibited a higher infiltration level of activated DCs, quiescent mast cells, and Tfh, whereas the infiltration of monocytes, CD8^+^ T cells, and Tregs was significantly lower (87). Furthermore, the high-risk group demonstrated a markedly elevated expression of key immune checkpoint molecules, correlating with poorer survival outcomes in patients with higher risk scores (87). These findings indicate that differences are strongly linked to clinical prognosis in immune cell infiltration (87). Ma also reported that UCEC patients harboring deep or shallow deletion alterations in m6A regulatory genes—including IGF2BP1, ZC3H13, METTL14, ALKBH5, and YTHDC1—exhibited worse prognoses, suggesting these genetic aberrations as potential m6A-related prognostic markers (88). Notably, the expression of ZC3H13, YTHDC1, and IGF2BP1 was positively correlated with PD-L1 expression in UCEC (88). Functional studies revealed that knockdown of ZC3H13 or YTHDC1 promoted the proliferation and invasion of UCEC cells (88). In summary, the study identified genetic alterations in m6A regulatory genes and revealed a clear association between reduced m6A levels and adverse clinical outcomes. These results have advanced our understanding of RNA epigenetic modifications and highlight m6A regulators involved in immune infiltration, disease progression, and therapeutic response as independent prognostic factors in UCEC. They provide novel molecular biomarkers and potential therapeutic targets for the management of UCEC.

In most cases, genetic information is transcribed into an extraordinarily complex RNA network, in which only 1% to 2% of transcripts are translated into proteins. Non-coding RNAs play a critical role in post-transcriptional regulation by modulating the transcriptional and translational activity of tumor-associated RNAs, thereby influencing tumor cell function and clinical outcomes. Pan conducted a pan-cancer genomic screening and identified that the expression of m6A-related lncRNAs LNCTAM34A was significantly downregulated in UCEC, correlating with poorer prognosis (89). Patients in the high-expression group of LNCTAM34A exhibited higher infiltration levels of immune cells, including activated DCs, B cells, and Tregs, as well as elevated expression of key immune checkpoint molecules, suggesting that LNCTAM34A may serve as a prognostic biomarker for UCEC (89). Additionally, Shan identified five m6A-related lncRNAs and constructed a risk score model to predict UCEC prognosis (90). Low-risk patients displayed higher immune scores compared to high-risk patients (90). The risk score was negatively correlated with resting DCs, neutrophils, activated NK cells, and activated mast cells but positively correlated with activated DCs, Tfh cells, and M1 macrophages, indicating a potential association between the risk score and dysregulation of the antigen-presenting system (90). Furthermore, patients in the high-risk group exhibited higher pathological grade and increased PD-L1 expression relative to those in the low-risk group (90). PD-L1 overexpression can protect tumor cells from CD8^+^ T -cell-mediated cytotoxicity, contributing to immune evasion (90). Zhou also developed a multivariate Cox proportional hazards regression model to stratify UCEC patients into high- and low-risk groups (91). In terms of immune function, the high-risk group showed heightened pro-inflammatory responses and IFN-I signaling compared to the low-risk group, suggesting a potential immunosuppressive role in UCEC progression (91). In another study, Zhao established a risk stratification model based on m6A methylation regulators, classifying UCEC patients into high- and low-risk groups according to their risk scores (92). The comprehensive analysis indicated that RNA modification regulators are closely associated with both patient prognosis and the TME in UCEC (92). Patients in the low-risk group exhibited lower levels of RNA modification, greater immune cell infiltration, and higher expression of immune checkpoint genes such as PDCD1 and CTLA4, suggesting enhanced responsiveness to immunotherapy (92). These findings confirm that the risk score is an independent predictor of clinical outcomes in UCEC patients.

In summary, the aforementioned studies have systematically investigated the impact of m6A RNA modification on the prognosis of patients with UCEC, thereby providing novel insights and robust foundational evidence for further exploration of the functional role of this epigenetic mechanism in UCEC. The integration of multiple biomarkers into a unified predictive model significantly enhances prognostic accuracy, enabling more precise prediction of tumor progression and the identification of reliable research directions for clinical treatment planning.

##### N1-methyladenosine modification

N1-methyladenosine (m1A) is a prevalent RNA modification that plays a significant role in tumorigenesis and progression. m1A-related lncRNAs (mRLs), owing to their high efficiency, tissue specificity, and molecular stability, have emerged as potential biomarkers and therapeutic targets for immunotherapy in UCEC patients. Liu identified two distinct molecular clusters based on the expression profiles of 28-mRLs (93). Cluster 2 was characterized by a lower proportion of Tregs and a higher proportion of M1 macrophages, yet it exhibited an overall reduced level of anti-tumor immune infiltration and diminished responsiveness to PD-1 and CTLA-4 blockade therapies (93). This cluster also displayed impaired intrinsic anti-tumor immunity and a poorer response to immunotherapy, suggesting an immunosuppressive TME (93). A prognostic model incorporating 10 mRLs was developed, revealing that patients had higher ESTIMATE scores, elevated levels of immune cell infiltration, increased IPS in the low-risk group, enhanced immune activation status, higher expression of immune checkpoint molecules, and greater tumor immunogenicity (93). These features indicate a favorable immune contexture, suggesting that such patients may benefit from ICI therapy to achieve improved survival outcomes. In contrast, the high-risk group showed reduced infiltration of CD8^+^ T cells, Tregs, γδ T cells, and activated NK cells, along with the increased presence of both M1 and M2 macrophages (93). These characteristics are indicative of T cell exhaustion and defective T-cell trafficking into the tumor bed, which are associated with immune evasion mechanisms (93). The observed reduction in immune infiltration and T cell dysfunction aligns with the definition of the “immune desert” phenotype—a state marked by the absence of effective anti-tumor immunity, poor response to ICI treatment, and compromised immune surveillance. Consequently, high-risk UCEC patients are classified as harboring “cold tumors,” which are permissive for immune escape (93). In summary, this mRL-based prognostic model enables an accurate prediction of clinical outcomes and holds promise as a tool for guiding personalized immunotherapeutic strategies in UCEC. It may serve as a novel framework for enhancing immunotherapy responsiveness, facilitating individualized treatment selection, and improving survival prognosis in advanced UCEC.

##### N7-methylguanosine modification

N7-methylguanosine (m7G) is one of the most prevalent RNA base modifications involved in post-transcriptional regulation. It plays a critical role in gene expression, RNA processing, metabolism, protein synthesis, and transcript stability throughout the mRNA life cycle. Chen identified nine m7G-related miRNAs associated with prognosis using the TargetScan database and constructed a risk score model in UCEC (94). These differentially expressed miRNAs were shown to be involved in regulating immune activity within the TME as well as in the initiation and progression of UCEC (94). Meanwhile, Zhao analyzed multi-omics data from UCEC samples in TCGA database and developed a prognostic risk model based on four m7G-related mRNAs (95). ssGSEA revealed that high-risk UCEC samples exhibited significantly increased infiltration of immune cells that promote tumor proliferation and metastasis, such as tumor-associated macrophages, while the expression of key immune checkpoint molecules was predominantly reduced (95). These findings suggest that the m7G-related mRNA risk model may serve as a predictive tool for the clinical efficacy of immune checkpoint blockade therapy in UCEC patients, indicating that poor prognosis may be linked to the accumulation of pro-tumorigenic immune cells in high-risk individuals (95). Furthermore, patients displayed higher TMB levels in the low-risk group, which is associated with improved clinical outcomes (95). This implies that low-risk and high-TMB patients may exhibit enhanced immunogenicity, making their tumors more recognizable to the immune system and potentially deriving greater benefit from ICI therapy (95). Collectively, these results enhance our understanding of the impact of m7G-related mRNAs on UCEC prognosis and response to immunotherapy, offering novel insights for therapeutic development and holding significant clinical potential for the design of UCEC-specific immune-targeted treatments.

##### Chromatin remodeling

Chromatin remodelers, as key upstream regulators in epigenetics, play a critical role in tumorigenesis and progression — for example, PHF6 is a highly conserved epigenetic transcriptional regulator involved in chromatin modification and plays a pivotal role in the precise regulation of gene expression during tissue homeostasis and embryonic development (96). In UCEC, PHF6 knockdown promotes T cell infiltration and suppresses tumor formation through enhanced immune surveillance in cancer cells (96). Conversely, a high expression of PHF6 is associated with reduced infiltration of CD8^+^ T cells and CD4^+^ T cells, along with aberrant activation of NK cells and M1 macrophages, resulting in diminished effective immune cell infiltration (96). These findings suggest that PHF6 may contribute to an immunosuppressive TME. Therefore, it is hypothesized that inhibition of PHF6 could enhance responsiveness to immunotherapy in UCEC patients, indicating that PHF6 may serve as a valuable biomarker for assessing immune cell abundance and participating in immune regulatory mechanisms in UCEC (96).

Wu identified nine TME-related chromatin remodeling regulators (CRRs) to construct a prognostic risk model (97). UCEC patients were stratified into low- CRR and high- CRR groups based on the median risk score, with those in the high- CRR group exhibiting poorer clinical outcomes (97). GSVA and GSEA revealed that pathways associated with cell proliferation and tumor growth were significantly activated in the high- CRR group, whereas immunity- and inflammation-related pathways were enriched in the low- CRR group (97). This suggests that differential immune response states may underlie the divergent prognoses observed between the two groups. Furthermore, CRRs were negatively correlated with stromal, immune, and ESTIMATE scores but positively correlated with tumor purity (97). Notably, samples displayed higher expression levels of chemokines and their receptors in the low- CRR group, which are critical for recruiting anti-tumor immune cells, including CD8^+^ T cells, DCs, and NK cells (97). These findings indicate that the high- CRR group represents a non-inflammatory tumor phenotype and is likely to exhibit poor responsiveness to ICB therapy (97). Additionally, the methylation levels of key CD8^+^ T effector signature genes—such as GZMA, GZMB, IFNG, CXCL10, and PRF1—as well as PD-1, were significantly reduced in the low- CRR group, while TMB was markedly elevated compared to the high- CRR group (97). Elevated TMB promotes the generation of mutation-derived neoantigens, thereby enhancing tumor immunogenicity and facilitating the activation of cytotoxic T lymphocytes (97). Thus, when the immune-related CRR is combined with TMB or MSI status, it demonstrates superior predictive performance for immunotherapeutic response (97). In summary, these comprehensive and in-depth studies elucidate the critical role of chromatin remodeling regulators in shaping the tumor immune microenvironment of UCEC. Their strong predictive value for both immunotherapy and chemotherapy responses highlights their potential as promising biomarkers for guiding the development of personalized and precision treatment strategies in UCEC.

A growing body of evidence indicates that lncRNAs can regulate gene expression at both transcriptional and epigenetic levels and play a significant role in chromatin remodeling. Enhancer RNA, a functional subclass of lncRNA transcribed from enhancer regions, interacts with target promoters to facilitate the transcriptional activation of downstream genes. Enhancer RNA (eRNA) is typically characterized as bidirectional non-coding transcripts. IGFBP7 antisense RNA1 (IGFBP7-AS1) is a corresponding lncRNA, which is positively correlated with its target gene IGFBP7 in tumor tissues (98). IGFBP7-AS1 has been identified as a key enhancer RNA with favorable prognostic value in UCEC (98). Studies have demonstrated that the higher expression levels of IGFBP7-AS1 and IGFBP7 are associated with increased stromal and immune cell infiltration, as well as reduced tumor purity, suggesting that patients with an elevated expression of these genes may exhibit enhanced responsiveness to immunotherapy (98). Furthermore, IGFBP7-AS1 and IGFBP7 are significantly correlated with various T cell subsets, including CD8^+^ T cells, Th cells, Tfh, Tγδ, Tregs, Th1 cells, and multiple immune checkpoint molecules such as PD-1 and CTLA-4 (98). Notably, the high expression of IGFBP7-AS1 and IGFBP7 is significantly associated with lower TMB, which may provide valuable insights for patient stratification in immunotherapy (98). These findings confirm that IGFBP7-AS1 is an immune-related eRNA that contributes to a more favorable tumor immune microenvironment and is linked to improved clinical outcomes.

In summary, these studies have established CR-related prognostic models that not only hold significant clinical value for the molecular classification and prognosis assessment of UCEC but also enhance our understanding of heterogeneity in the tumor immune microenvironment. The models offer a valuable reference for evaluating patient prognosis subtypes and immune status and lay a theoretical foundation for the development of prospective molecular biomarkers in UCEC.

##### Histone modification

Kinases are enzymes that catalyze the transfer of phosphate groups from high-energy donor molecules, such as ATP, to specific target molecules (substrates)—a process known as phosphorylation. Protein kinases are localized in various subcellular compartments, including the nucleus, mitochondria, microsomes, and cytoplasm. By catalyzing the covalent attachment of phosphate groups to hydroxyl residues of specific serine, threonine, or tyrosine amino acids within target proteins, they modulate protein conformation, activity, and functional interactions, thereby regulating essential cellular processes such as signal transduction, cell cycle progression, apoptosis, and gene expression. Consequently, dysregulation of protein kinase function—through mechanisms such as overexpression, aberrant subcellular localization, point mutations, or disruptions in upstream signaling pathways—can contribute to the initiation and progression of numerous diseases, particularly cancer.

For instance, TK1 and CKS2 are key kinases involved in cell cycle regulation. TK1 is a critical enzyme that catalyzes the phosphorylation of thymidine to thymidine monophosphate, a rate-limiting step in the salvage pathway of DNA synthesis, where thymidine serves as an essential precursor (99). TK1 expression is negatively correlated with the infiltration levels of CD8^+^ T cells, macrophages, and DCs, suggesting impaired antigen presentation and cytotoxic lymphocyte activity in the tumor microenvironment, which may contribute to immune evasion—a potential mechanism underlying the poor prognosis observed in UCEC patients with high TK1 expression (99). CKS2, a member of the cyclin-dependent kinase regulatory subunit family, plays a pivotal role in cell cycle progression in somatic cells and early embryonic development (100). In UCEC, CKS2 is significantly overexpressed, and its upregulation is strongly associated with altered infiltration levels of B cells, CD4^+^ T cells, and neutrophils (100). It may influence tumorigenesis and disease progression through multiple biological pathways, highlighting its potential as a promising clinical biomarker for UCEC (100). BUB1 and its paralog BUB1B are core components of the spindle assembly checkpoint complex. These proteins play essential roles in preventing premature sister chromatid separation and maintaining chromosomal stability, thereby reducing the incidence of aneuploidy (101). Zhang reported that both BUB1 and BUB1B are significantly upregulated in UCEC, and high expression levels are associated with reduced OS and PFS (101). Studies further demonstrate that BUB1 and BUB1B exhibit strong correlations with the abundance of various TILs and are significantly linked to the expression of multiple immunosuppressive molecules and immune agonists, suggesting their involvement in modulating tumor immune infiltration and potentially mediating immune escape mechanisms in UCEC (101). Wei constructed a prognostic model comprising seven kinases and stratified the patients into high-risk and low-risk groups based on risk scores (102). Survival analysis revealed that patients in the high-risk group had significantly lower overall survival rates compared to those in the low-risk group (102). According to CIBERSORT algorithm-based deconvolution, the low-risk group exhibited higher infiltration levels of multiple anti-tumor immune cell populations, including CD8^+^ T cells, Th1 cells, and Th2 cells, and showed elevated activity in immune-related pathways such as HLA signaling and T cell co-stimulation (102). This suggests that attenuated anti-tumor immunity may be a contributing factor to their adverse clinical outcomes in high-risk patients (102). In summary, these kinases and kinase-based prognostic models demonstrate independent predictive capacity for UCEC diagnosis and response to immunotherapy. They offer valuable insights and strategic breakthroughs for the development of kinase-targeted therapies and support the construction of reliable, genome-wide comprehensive models for precision prognosis prediction in UCEC.

SUMOylation is a reversible post-translational modification of histones that plays a critical role in various biological processes, including cell cycle regulation, signal transduction, and DNA damage repair. This modification is conserved across nearly all eukaryotic organisms and is tightly regulated by a cascade of SUMOylation-related enzymes, namely, the SUMO-activating enzyme E1, the SUMO-conjugating enzyme E2, and SUMO ligase E3 (103). Mature SUMO proteins are initially activated by E1 enzymes and subsequently conjugated to specific substrate proteins through the coordinated actions of E2 and E3 enzymes (103). The deSUMOylation process is mediated by SENPs (SUMO-specific proteases), which regulate the dynamic equilibrium of SUMOylation (103). Furthermore, SUMOylation interacts with other post-translational modifications—such as phosphorylation, ubiquitination, methylation, and acetylation—thereby contributing to a complex and finely tuned protein regulatory network (103). Lei compared the expression of 20 SUMOylation-related genes between normal and cancerous endometrial tissues and identified three genes to construct a prognostic risk model (103). The resulting risk score was determined to be an independent predictor of poor survival in UCEC. Functional analyses revealed higher levels of immune cell infiltration and enhanced activity of immune-related pathways in the low-risk group, suggesting that the unfavorable prognosis observed may be associated with impaired anti-tumor immunity in the high-risk group (103). In conclusion, SUMOylation-related genes play a significant role in tumor immune regulation, and this study could provide a valuable foundation for future investigations into their functional roles in UCEC.

#### Inflammation–cancer transformation

MGST1 functions as a critical inflammatory mediator with glutathione S-transferase and peroxidase activities. Yan demonstrated that MGST1 exhibits strong connectivity with proteins involved in glutathione metabolism, electron transfer, and oxidative stress responses by PPI network analysis—such as LAMP1, LAMP2, STOM, and VNN1. MGST2 and MGST3 are also members of the glutathione peroxidase family and are implicated in chemotherapy-induced oxidative stress and oxidative DNA damage (21). Notably, overexpression of MGST1 has been shown to confer resistance to ferroptosis in UCEC cells, highlighting its essential role in tumor initiation and progression (21). Thus, MGST1 may serve as a potential prognostic biomarker for UCEC patients (21). Gu developed a robust prognostic risk model based on five hub inflammation-related long non-coding RNAs (IRLs), which was validated as an independent prognostic factor in UCEC (104). The transcriptional profile of these IRLs was significantly associated with immune cell infiltration, tumor purity, and overall immune status (104). By characterizing the immune landscape between the two risk groups, the study found that the risk score was negatively correlated with the immune score, suggesting that high-risk patients are more likely to exhibit an immunosuppressive tumor microenvironment (104). CIBERSORT analysis further revealed increased infiltration of M2 macrophages in the high-risk group (104). Given that M2 macrophages represent an immunosuppressive subset of tumor-associated immune cells, their elevated presence is closely linked to poor clinical outcomes in UCEC (104). The aforementioned studies have successfully identified novel inflammatory phenotypes of UCEC and established corresponding prognostic models, which can be utilized to assess patient survival outcomes and inform clinical treatment decisions. By reflecting the underlying immune landscape and evaluating immune response to therapy, these models provide critical insights into immunotherapeutic efficacy and support the development of personalized chemotherapy strategies. The findings thus offer valuable references for future research and hold promise for improving individualized clinical management in UCEC.

#### Traditional Chinese medicine

In China, TCM has been widely accepted as an alternative therapeutic approach for tumors. Accumulating evidence indicates that TCM exerts a holistic and systemic regulatory effect on disease processes, demonstrating significant efficacy in the control and prevention of cancer. In contrast to conventional anti-cancer drugs, which are often associated with pronounced adverse effects, numerous natural small-molecule compounds derived from Chinese medicinal herbs exhibit potential tumor selectivity and low cytotoxicity. These properties have garnered substantial attention in the field of oncology drug development. Representative bioactive compounds include puerarin and luteolin, which have been extensively studied for their anti-tumor activities and underlying mechanisms.

Puerarin is a small-molecule isoflavone glycoside extracted from *Pueraria lobata*. Lin employed network pharmacology and bioinformatics analyses to construct a comprehensive database of drug targets and disease-associated genes (105). Two prognostic genes were subsequently screened to stratify patients into high- and low-risk groups, respectively, with the low-risk group demonstrating a significantly higher survival rate than the high-risk group (105). The findings suggest that puerarin may exert its anti-cancer effects by modulating the immune response of endometrial immune cells through the “LPS” and “bacterial molecule” signaling pathways (105). Puerarin inhibits the activation of the MAPK pathway, which may represent a key mechanism underlying its suppression of UCEC cell proliferation (105). Further studies are warranted to elucidate the detailed molecular mechanisms and clinical potential of puerarin in cancer therapy (105). Luteolin is a natural flavonoid compound widely present in traditional Chinese medicine. It possesses potent antioxidant and anti-inflammatory properties and has been shown to inhibit tumor cell proliferation and metastasis, contributing to its anti-neoplastic potential (106). Zhao constructed a luteolin-related genetic prognostic model for UCEC (106). The enrichment analyses revealed significant enrichment in biological processes related to “oxidative stress response” and the “IL-17 signaling pathway” (106). It was hypothesized that luteolin may attenuate UCEC progression by suppressing the production of inflammatory mediators such as IL-17, alleviating oxidative stress, and downregulating the expression of key molecules including MMP1, IL-17, and VEGF (106). These effects may collectively inhibit cancer cell migration and tumor angiogenesis (106). Furthermore, luteolin might suppress ROS generation and inflammatory mediator release, thereby modulating downstream signaling pathways and altering the tumor-promoting microenvironment, ultimately inhibiting UCEC growth (106). In conclusion, these bioactive components derived from traditional Chinese medicine have expanded the therapeutic landscape for UCEC, offering new options for patient management and establishing a theoretical foundation for the development of innovative treatment strategies against this malignancy.

## Discussion

### Biological activity of overlapping genes

Human tumors are widely recognized as a major global health challenge due to their high incidence and mortality rates. Although innovative therapeutic approaches—such as chemotherapy, radiotherapy, immunotherapy, and targeted therapy—have demonstrated promising clinical efficacy, the prognosis and survival outcomes for patients with advanced-stage disease remain unsatisfactory. In particular, an increasing number of studies focus on discovering predictive biomarkers that can distinguish patient subgroups likely to benefit from immunotherapy from those who are non-responsive, thereby enabling the development of precise and individualized treatment strategies. This review summarizes recent advances in immune-related biomarkers identified through high-throughput sequencing technologies and bioinformatics analyses, compiling a comprehensive list of functionally diverse targets implicated in the initiation and progression in UCEC. From this dataset, frequently reported bioactive molecules were selected based on recurrence across multiple studies, including genes such as RAC3, PGR, CDKN2A, SIX1, and TP53. These molecules were observed to appear at least three times in the analyzed literature, suggesting their central regulatory roles. In-depth investigation of these recurrently identified factors may enhance our understanding of the tumor microenvironment and the immunopathological mechanisms driving UCEC, offering a solid theoretical foundation and prioritized candidate targets for future immunotherapeutic interventions and vaccine development.

RAC is a subgroup of the Ras superfamily of GTPases and belongs to the Rho GTPase family. They include ubiquitously expressed RAC1, hematopoietic cell-specific RAC2, and neuron-enriched RAC3 (9). RAC proteins play critical roles in regulating actin cytoskeleton remodeling, cell survival, stem cell development, and tumor progression (9). In UCEC, RAC3 is involved in modulating catalytic enzyme activity and chromatin remodeling processes, promoting malignant behaviors such as proliferation and invasion by mediating tumor immune infiltration (9). Multiple studies have demonstrated that RAC3 drives the expression of downstream genes to enhance tumor stemness and facilitate invasion and metastasis in colorectal adenocarcinoma, promotes extracellular matrix proteolysis and invasive capacity in ovarian adenocarcinoma, induces cell invasion and metastasis through the regulation of adhesion and matrix degradation in breast cancer, and enhances the migration, invasion, and EMT of lung adenocarcinoma cells via activation of the p38 MAPK signaling pathway (9) (**Figure 1**). The PGR is a key mediator of tumor response to progesterone therapy, regulating cellular proliferation and differentiation through the modulation of gene transcription (107). Upon binding to progesterone, PGR functions as a transcription factor that regulates autophagy-related genes, participates in lipid metabolism and ferroptosis, and modulates estrogen signaling and associated metabolic pathways (107). However, reduced PGR expression leads to the downregulation of ATG7 transcription, resulting in impaired autophagy and contributing to disease progression in UCEC (107). In hormone-dependent cancers such as UCEC and breast cancer, PGR positivity is generally associated with favorable responses to hormonal therapy (107). Downregulation of PGR diminishes the efficacy of progesterone treatment and contributes to the development of therapeutic resistance (107). In progesterone-insensitive UCEC patients, heme metabolism is upregulated, leading to increased release of heme, which promotes polarization of macrophages (Mφs) toward an M2-like phenotype, thereby fostering tumor immune tolerance (107). Furthermore, heme suppresses the expression of the protective cytokine IL-33, resulting in elevated levels of PAX8. PAX8 binds to the PGR promoter and acts as a transcriptional repressor, inhibiting PGR expression (107). Future research should further investigate the intrinsic mechanisms underlying progesterone resistance to identify novel therapeutic targets and improve clinical management strategies in UCEC (107) (**Figure 1**). CDKN2A, located on chromosome 9p21, is a member of the INK4 family of cyclin-dependent kinase inhibitors and encodes multiple protein isoforms, including p14ARF and p16INK4a (108). It plays a pivotal role in cell cycle regulation and functions as a tumor suppressor by inhibiting uncontrolled proliferation and tumorigenesis (108). In UCEC, CDKN2A contributes to pro-tumorigenic immune infiltration and drives tumor initiation, progression, and therapeutic resistance by participating in key biological processes such as lipid metabolism, ferroptosis, oxidative stress, and EMT (108) (**Figure 1**). These findings have been corroborated in other malignancies, including non-small cell lung cancer, hepatocellular carcinoma, renal cell carcinoma, and triple-negative breast cancer, highlighting its potential as a biomarker and immunotherapeutic target in oncology (108). Accumulating evidence indicates that overexpression of the transcription factor SIX1 promotes tumorigenesis and activates cancer cell proliferation, metastasis, and EMT (109). SIX1 not only regulates immune cell infiltration within the tumor microenvironment but also interacts with lncRNA LINC02936 in UCEC, which enhances ceruloplasmin expression, reduces intracellular Fe²^+^ and ROS levels, and suppresses ferroptosis, thereby facilitating tumor progression (109). Additional studies have confirmed that SIX1 overexpression amplifies oncogenic signaling cascades across various cancers, including breast, hepatocellular, ovarian, cervical, pancreatic, and lung adenocarcinomas (69) (**Figure 1**). In 2013, TCGA published a comprehensive genomic classification, defining four molecular subtypes: POLE-mutant, dMMR, CN-L), and CN-H in UCEC. Among these, the CN-high subtype is characterized by a high TP53 mutation rate (approximately 90%) and a low PTEN mutation rate (11%) (110). A meta-analysis data from six studies comparing TCGA and ProMisE classification systems revealed that the patients with TP53 mutations or aberrant p53 expression exhibit the poorest OS in UCEC, with a significantly reduced 5-year survival rate of approximately 54%, which is markedly lower than the other three subtypes (110) (**Figure 1**). Collectively, this molecular stratification system demonstrates strong prognostic value and holds significant implications for guiding personalized therapeutic decisions in UCEC (**Figure 1**).

### Prognostic value of overlapping genes

**Figure 3:**
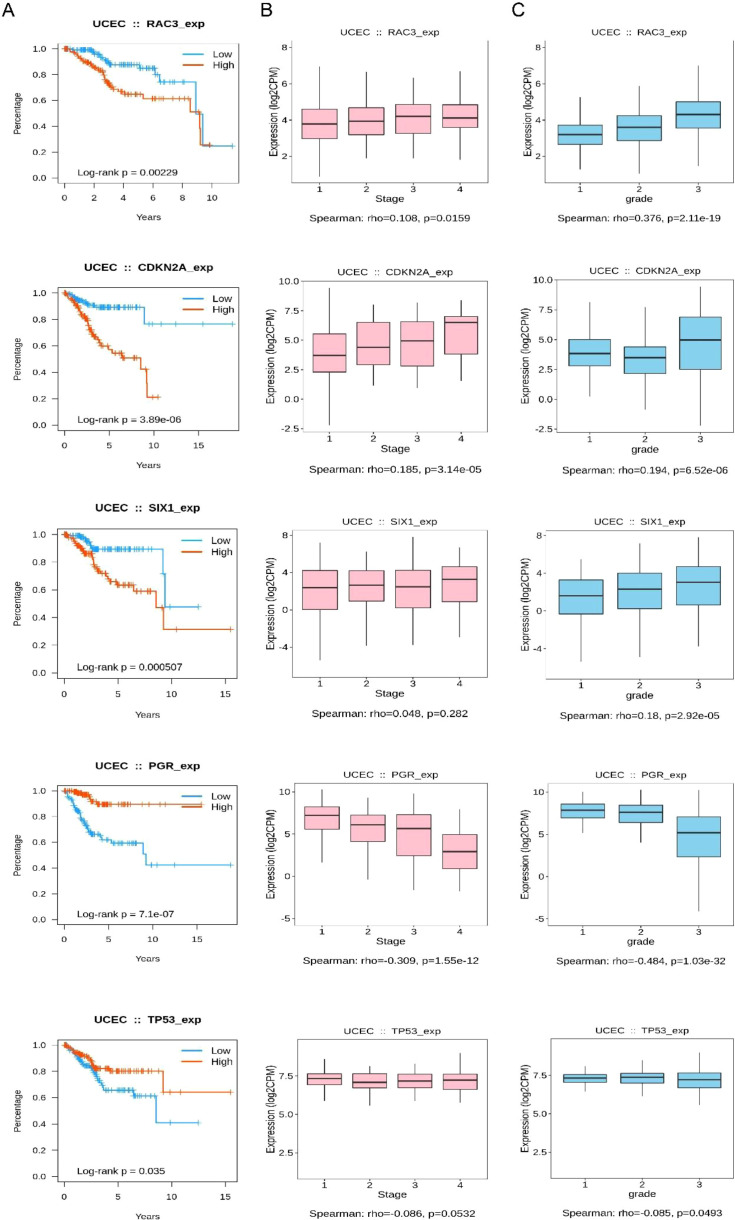
Survival analysis of high-frequency overlapping genes. **(A)** Overall survival. **(B)** Association with FIGO stage. **(C)** Association with histological grade.

We first retrieved clinical and molecular data for UCEC from TCGA database. Using SangerBox (http://sangerbox.com), an open-access bioinformatics platform, we performed survival analysis and generated Kaplan–Meier curves to assess the association between the expression levels of frequently altered genes—including RAC3, PGR, CDKN2A, SIX1, and TP53—and OS. Additionally, we evaluated correlations between these gene expression levels and key clinicopathological parameters, namely, FIGO stage and histological grade. These analyses were conducted to systematically investigate the prognostic relevance of the selected genes and to evaluate their potential utility as biomarkers for risk stratification in endometrial carcinoma. The results demonstrated that all five genes were significantly associated with OS in UCEC, with statistically significant *p*-values. Specifically, the high expression of RAC3 (*p* = 0.00229), CDKN2A (*p* = 3.89e-06), and SIX1 (*p* = 0.000507) was negatively correlated with OS, indicating that elevated expression levels are associated with poorer prognosis. In contrast, the higher expression of PGR (*p* = 7.1e-07) and TP53 (*p* = 0.035) was positively correlated with improved OS, suggesting a protective role in patient outcomes. Additionally, we investigated the associations between these five genes and clinical parameters, including FIGO stage and histological grade. RAC3 (*p* = 0.0159, *R* = 0.108) and CDKN2A (*p* = 3.14e-05, *R* = 0.185) showed positive correlations with advanced FIGO stage, whereas PGR (*p* = 1.55e-12, *R* = -0.309) exhibited a significant negative correlation, implying that a lower PGR expression is linked to more advanced diseases. No statistically significant associations were observed between SIX1 (*p* = 0.282, *R* = 0.048) or TP53 (*p* = 0.0532, *R* = -0.086) and FIGO stage. Similarly, for histological grade, RAC3 (*p* = 2.11e-19, *R* = 0.376), CDKN2A (*p* = 6.52e-06, *R* = 0.194), and SIX1 (*p* = 2.92e-05, *R* = 0.18) were positively correlated, indicating their association with higher tumor differentiation grades, while PGR (*p* = 1.03e-32, *R* = -0.484) and TP53 (*p* = 0.0493, *R* = -0.085) were negatively correlated, consistent with a favorable prognostic impact (**Figure 3**). These findings collectively confirm that the elevated expression of RAC3, CDKN2A, and SIX1 serves as a risk factor for adverse outcomes, whereas high PGR expression is associated with better prognosis in UCEC. Furthermore, wild-type TP53 appears to function as a protective factor, reinforcing its tumor-suppressive role. This consistency across survival and clinicopathological analyses validates the accuracy and reliability of these immune-related genes as prognostic biomarkers in UCEC. These genes may therefore be valuable in assessing long-term survival and treatment response in patients. In future studies, the specific molecular mechanisms underlying their functions and their potential utility as targets for immunotherapeutic interventions should be further elucidated. Once their prognostic value is consistently validated in independent cohorts, a gene-based immune scoring system incorporating these markers could be developed for risk stratification—paralleling the established T-cell inflamed gene signature used in colorectal cancer—which would enhance precision oncology and support individualized clinical decision-making.

### Limitations

Although the review hold substantial clinical relevance and offer promising avenues for future research in UCEC, several limitations warrant careful consideration. First, the publicly available databases contain a limited and non-comprehensive set of clinicopathological parameters. To robustly validate the clinical utility and reliability of the identified immune markers, prospective, multicenter studies involving larger, well-annotated patient cohorts are essential. Second, the current analysis relies exclusively on bulk transcriptomic and associated clinical data. Complementary molecular data—including copy number alterations and other epigenomic or genomic features—are critically involved in UCEC and should be integrated into future mechanistic investigations. Third, the diagnostic and predictive performance of any single biomarker remains inherently constrained. Therefore, future efforts should prioritize the development and validation of multimodal biomarker panels—comprising diverse molecular entities—alongside advanced computational algorithms optimized for clinical discrimination. Notwithstanding these limitations, this study contributes novel candidate immune targets for endometrial cancer immunotherapy and lays a foundational framework for the rational design of next-generation therapeutic strategies.

## Conclusion

This study systematically reviewed a comprehensive body of bioinformatics literature related to immune infiltration in UCEC. Key gene markers and predictive models involved in critical biological processes—such as metabolic regulation, programmed cell death, mitophagy, endoplasmic reticulum stress, and inflammation-to-cancer transition—were identified and characterized. These bioactive molecules mediate the tumor immune microenvironment through distinct molecular mechanisms and signaling pathways, enabling the screening of numerous potential immune biomarkers and therapeutic targets. An integrated analysis of these candidates offers novel and insightful perspectives for UCEC research, contributing to a deeper understanding of its epigenetic regulation and immunotherapeutic potential. Elucidating their roles establishes a foundational framework for deciphering the functional interplay between these genes and anti-tumor immune responses in immune infiltration, thereby facilitating the development of personalized treatment strategies for the patients. Furthermore, this study identified five high-frequency immune-related genes reported across multiple independent studies. Targeting these recurrently implicated genes represents a promising therapeutic approach to disrupt key signaling pathways driving UCEC progression, providing a robust basis for future translational research in targeted immunotherapy. In addition, the anti-cancer properties of active components derived from traditional Chinese medicine—including luteolin, quercetin, and puerarin—were highlighted. Future efforts should focus on developing a multidimensional and integrative treatment strategy that combines conventional modalities such as radiotherapy and chemotherapy with immunotherapy, hormone therapy, and traditional Chinese medicine. Such a comprehensive approach holds significant promise for improving clinical outcomes and survival prospects for patients with UCEC.
