# Supplementary material for: Recent advances in characterizing the immune microenvironment and biomarkers of endometrial carcinoma
Source: Front Immunol. 2026 May 29;17:1779211. doi: 10.3389/fimmu.2026.1779211 (PMC13260069; doi:10.3389/fimmu.2026.1779211)
Supplement: Supplementary file 1 [file Table1.docx]

| Function | Reference | Year | Tumor Immunology | Gene |
| --- | --- | --- | --- | --- |
| Chromatin Remodeling | Wang^[96]^ | 2023 | Immune Infiltration | PHF6 |
|  | Wu^[97]^ | 2023 | Immune Assessment  Immunotherapy | FOXP3、APOBEC3G、CUL4B、RAC3、HJURP、SCML2、HMGB3、TSPYL5、ZBTB16 |
| CRGs | Ran^[28]^ | 2023 | Cuproptosis  Glycometabolism  Immune Infiltration | FDX1 |
|  | Chen^[29]^ | 2022 | Immune Infiltration | FDX1、LIAS、DLAT、CDKN2A、LIPT1、DLD、PDHB、MTF1、GLS |
|  | Lin^[30]^ | 2023 | Cell Adhesion  Immune Infiltration | GLS、CDKN2A、PC、SUCLG1 |
| DNA damage repair-related genes | Lu^[80]^ | 2023 | Immune Infiltration  Immunotherapy | MSH2 |
|  | Zhou^[81]^ | 2024 | Immunotherapy | MSH6 |
|  | Yang^[83]^ | 2024 | Radiosensitivity  Immunotherapy | PTEN、PIK3CA、ARID1A、P53 |
| DNA Methylation | Liu^[84]^ | 2021 | Immune Infiltration | RILPL2 |
|  | Cai^[85]^ | 2023 | Immune Infiltration | SYTL1 |
|  | Liao^[86]^ | 2024 | Immune Infiltration | SPC25 |
| DEERGs | Li^[50]^ | 2022 | Immune Response Prediction | PKP3、NMU、 TJP3、PTPN6、BATF、GAL、HPRT1、NRIP1、ASS1、LARGE1、ANXA9、DNAJC12、PGR |
| Enhancer RNA | Liu^[98]^ | 2022 | Immune Infiltration | IGFBP7-AS1 |
| Enzymes | Zhu^[14]^、Yu^[15]^ | 2024 | Immunotherapy  Glycolysis | GGH |
|  | Yan^[21]^ | 2022 | Ferroptosis  Immune Infiltration | MGST1 |
|  | Wu^[59]^ | 2023 | Immune Infiltration | MTHFD2 |
|  | Zhang^[60]^ | 2023 | Immune Infiltration | PHGDH |
|  | Wang^[61]^ | 2023 | Immune Infiltration | PSAT1 |
|  | Su^[66]^ | 20242024 | Immune Infiltration  Immunotherapy  Immunophenotype Group | the MMP family |
| Endoplasmic reticulum stress-related genes (ERGs) | Zhou^[45]^ | 2023 | Immunotherapy | ATP2C2、CIRBP、CRELD2、DRD2 |
|  | Zhang^[46]^ | 2023 | Immune Escape  Immunotherapy | TRIB3、CREB3L3、XBP1、PPP1R15A |
| EMT-related genes (ERGs) | Liu^[76]^ | 2022 | Immunocompetence | FBN1、HIC1、SFRP4、COL11A1、ONECUT2、HOXB9、DLX4、MSX1、TNF、SIX1 |
|  | Ruan^[77]^ | 2022 | Immune Infiltration | EPHB2、TUFT1、CDKN2A、ONECUT2、RBP2、KLF8、E2F1、SIX1、ERBB2 |
| GLRGs | Lin^[4]^ | 2024 | Immunophenotype Group | AUP1、ESR1、ERLIN2、ASS1、OGDH、BCKDHB、SLC16A1、LPCAT1、HK2、PGR-AS1 |
| GPRs | Lei^[53]^ | 2022 | Tumor-Associated Inflammation  Immune Infiltration | Adhesion GPCRs |
|  | Chen^[54]^ | 2023 | Tumor Immune Microenvironment | P2RY14 |
| Histone Modification | Lei^[103]^ | 2023 | Immune Infiltration | SUMO4、PIAS4、SENP2 |
| Hypoxia-related DEGs | Jiao^[3]^ | 2023 | Immunotherapy | HOXB9、IL6、NR3C1、SRPX |
| Kinases | Sun^[99]^ | 2024 | Immune Infiltration | TK1 |
|  | Gao^[100]^ | 2022 | Immune Infiltration | CKS2 |
|  | Zhang^[101]^ | 2024 | Immune Infiltration  Immune Escape | BUB1、BUB1B |
|  | Wei^[102]^ | 2022 | Immune Infiltration | ALPK2、CAMKV、TTK、PTK6、MAST1、CIT、FAM198B |
| LMRGs | Shi^[1]^ | 2023 | Metabolic Energy  Immune Infiltration | TP53、MRPL3、NDUFA11、NAXE、CYP27A1、PDSS1、RNASEH1、ACAT2、MECP2、NDUFB9、PDHA1、CARS2、HPDL、ATPAF2、TIMM50、FBP1、ALDOB、NDUFA6 |
| LMRGs-FARs | Yang^[13]^ | 2023 | Immunotherapy | CDKN1A、ESR1、PGR、CDKN2A、PSAT1、RSAD2 |
| Mitophagy | Zhu^[47]^ | 2022 | Immune Infiltration  Immune Response Prediction | TIMM8A |
| m6A RNA modification | Pang^[87]^ | 2021 | Immune Infiltration | IGF2BP1、YTHDF3 |
|  | Ma^[88]^ | 2021 | Immune Infiltration | IGF2BP1、ZC3H13、METTL14、ALKBH5、YTHDC1 |
|  | Zhao^[92]^ | 2023 | Immune Infiltration  Immunotherapy | INSM1、SLC6A11、GDPD2、IGSF1 、KLRG2、GFRA4、DDC、TM4 SF20 、NRXN1、GYPA 、DLGAP3、AGMO 、WFDC10A |
| m7G RNA modification | Zhao^[95]^ | 2022 | Immune Infiltration  Immunotherapy | NSUN2、NUDT3、LARP1、NCBP3 |
| NMRGs | Hu^[6]^ | 2024 | DNA Replication  Proteasome  Mismatch Repair | SLC22A13、CYP8B1、NMRK1、NAXE、NT5E、NT5M |
| OSRGs | Li^[36]^ | 2023 | Immunotherapy | SLC7A11 |
|  | Liu^[37]^ | 2022 | Immune Infiltration | H3C1、CDKN2A、STK26、TRPM2、E2F1、CHAC1、MSX1 |
| OXPHOS | Liu^[5]^ | 2022 | Immune Infiltration | ATP5IF1、COX6B1、FOXP3、NDUFB11 |
| Proliferation and Metastasis | Pei^[67]^ | 2022 | Immune Infiltration | MCM4 |
|  | Li^[68]^ | 2021 | Immunoregulation | MAL |
|  | Zhao^[69]^ | 2023 | Immune Infiltration | SIX1 |
|  | Li^[70]^ | 2019 | Activation of TAMs | CTHRC1 |
|  | Wang^[71]^ | 2023 | Immune Infiltration  Ion Exchange | CLCN4 |
|  | Chen^[72]^ | 2024 | Immune Infiltration  Immunotherapy | SPAG5 |
|  | Jiang^[73]^ | 2024 | Immune Infiltration | ZDHHC1 |
|  | Geng^[74]^ | 2020 | Immune Infiltration | ZBTB7A |
|  | Wu^[75]^ | 2023 | Immune Infiltration | DCL1 |
| Rho GTPase family | Cai^[9]^  Huang^[58]^ | 20222023 | Immunoregulation | RAC3 |
| RRGs | Geng^[48]^ | 2022 | Immune Infiltration | ACAP1、ODF2、RBBP7、PHKA1、COG5、NRIP1、ZNF264、SOX12 |
|  | Liu^[49]^ | 2023 | Immunotherapy | CYBA、SMPD3 |
| SHMRGPI | Duan^[52]^ | 2023 | Immune Infiltration  Immunotherapy | ESRRB、KDM1A、HSD3B1、PGR、AKR1C3、ARSB、RDH8、KDM5B |
| TCM | Zhao^[106]^ | 2023 | Inflammation  Oxidative Stress | PRSS1、MMP1、ERBB2、NUF2 |
|  | Lin^[105]^ | 2022 | Immunotherapy | PIM1、BIRC5 |
| Transcription Factors | Darmawi^[55]^ | 2022 | Immune Infiltration | BHLHE22 |
|  | Chen^[56]^ | 2023 | Cell Cycle  Immune Infiltration | FOXM1 |
|  | Lu^[57]^ | 2022 | Immunotherapy | the IRF family |
| Tumor Angiogenesis | Nong^[78]^ | 2021 | Immune Infiltration | ANGPT1 |
|  | Wang^[79]^ | 2022 | Immune Infiltration | ATAD2 |
