# Supplementary material for: Recent advances in characterizing the immune microenvironment and biomarkers of endometrial carcinoma
Source: Front Immunol. 2026 May 29;17:1779211. doi: 10.3389/fimmu.2026.1779211 (PMC13260069; doi:10.3389/fimmu.2026.1779211)
Supplement: Supplementary file 2 [file Table2.docx]

**Supplementary table. 2** Abbreviations.

| CAF | cancer-associated fibroblast |
| --- | --- |
| ceRNA | competing endogenous RNA |
| CN-H | copy number-high |
| CN-L | copy-number low |
| CNV | copy number variation |
| CRs | chromatin regulatory factors |
| CRG | copper-related gene |
| CRL | cuproptosis-related lncRNA |
| CRR | chromatin remodeling regulator |
| DAMP | damage-associated molecular pattern |
| DC | dendritic cell |
| DDR | DNA damage repair |
| DEERG | estrogen response-related gene |
| DEG | differentially expressed gene |
| dMMR | mismatch repair deficiency |
| DRL | DDR-related long non-coding RNA |
| DSS | disease-specific survival |
| ECM | extracellular matrix |
| EMT | epithelial-mesenchymal transition |
| ER | endoplasmic reticulum |
| ERG | EMT-related gene |
| ESRRA | estrogen-related receptor alpha |
| FASN | fatty acid synthase |
| Fe-S | iron-sulfur |
| FRG | ferroptosis-related gene |
| GAP | GTPase-activating protein |
| GGH | γ-Glutamyl hydrolase |
| GLRG | glucose- and lipid metabolism-related gene |
| GLS | glutaminase |
| GO | Gene Ontology |
| GPCR | G protein-coupled receptor |
| GSEA | Gene Set Enrichment Analysis |
| GSVA | Gene Set Variation Analysis |
| HLA | human leukocyte antigen |
| ICB | immune checkpoint blockade |
| ICI | immune checkpoint inhibitor |
| iDC | immature dendritic cell |
| IFN-I | type I interferon |
| IFN-II | type II interferon |
| IHC | immunohistochemistry |
| IRG | iron-related gene |
| IRL | inflammation-related long non-coding RNA |
| IPS | immune phenotype score |
| KEGG | Kyoto Encyclopedia of Genes and Genomes |
| LDH | lactate dehydrogenase |
| LMRG | Lactate metabolism-related gene |
| LMRGs-FAR | lipid metabolism and ferroptosis-associated gene |
| lncRNA | long non-coding RNA |
| MDSC | myeloid-derived suppressor cell |
| miRNA | microRNA |
| MLKL | mixed lineage kinase domain-like pseudokinase |
| MMP | matrix metalloproteinase |
| MMR | mismatch repair |
| mRL | m1A-related lncRNA |
| mRNA | messenger RNA |
| MSI | microsatellite instability |
| MSI-H | microsatellite instability-high |
| MSS | microsatellite stable |
| NAD+ | nicotinamide adenine dinucleotide |
| NRL | necroptosis-related lncRNA |
| NK cell | natural killer cell |
| OS | overall survival |
| OSRG | oxidative stress-related gene |
| OXPHOS | oxidative phosphorylation |
| pDC | plasmacytoid dendritic cell |
| PFS | progression-free survival |
| PGR | progesterone receptor |
| PPI network | protein-protein interaction network |
| RIPK1 | receptor-interacting protein kinase 1 |
| ROS | reactive oxygen species |
| RR | radiotherapy-resistant |
| RRG | redox-related gene |
| RS | radiotherapy-sensitive |
| SHMRGPI | hormone metabolism-related gene prognostic index |
| ssGSEA | Single-sample gene set enrichment analysis |
| TAM | tumor-associated macrophage |
| TCA cycle | tricarboxylic acid cycle |
| TCGA | The Cancer Genome Atlas |
| Tcm | central memory T cell |
| TCM | traditional Chinese medicine |
| Tgd | γδ T cell |
| Th cell | T helper cell |
| TIDE | tumor immune dysfunction and exclusion |
| TIIC | tumor-infiltrating immune cell |
| TIL | tumor-infiltrating lymphocyte |
| TIME | tumor immune microenvironment |
| TMB | tumor mutation burden |
| TME | tumor microenvironment |
| TNF | tumor necrosis factor |
| Treg | regulatory T cell |
| UCEC | uterine corpus endometrial carcinoma |
| VEGF | vascular endothelial growth factor |
| WB | western blot |
